# Activity and Metabolic Versatility of Complete Ammonia Oxidizers in Full-Scale Wastewater Treatment Systems

**DOI:** 10.1128/mBio.03175-19

**Published:** 2020-03-17

**Authors:** Yuchun Yang, Holger Daims, Yang Liu, Craig W. Herbold, Petra Pjevac, Jih-Gaw Lin, Meng Li, Ji-Dong Gu

**Affiliations:** a Laboratory of Environmental Microbiology and Toxicology, School of Biological Sciences, The University of Hong Kong, Hong Kong SAR, Hong Kong, People’s Republic of China; b Shenzhen Key Laboratory of Marine Microbiome Engineering, Institute for Advanced Study, Shenzhen University, Shenzhen, People’s Republic of China; c Institute of Environmental Engineering, National Chiao Tung University, Hsinchu City, Taiwan; d University of Vienna, Centre for Microbiology and Environmental Systems Science, Division of Microbial Ecology, Vienna, Austria; e University of Vienna, The Comammox Research Platform, Vienna, Austria; f Joint Microbiome Facility of the Medical University of Vienna and the University of Vienna, Vienna, Austria; University of California, Santa Barbara; University of Southern California

**Keywords:** comammox *Nitrospira*, cyanase, full-scale WWTPs, homoacetate fermentation, metabolic versatility

## Abstract

The recent discovery of complete ammonia oxidizers (comammox) contradicts the paradigm that chemolithoautotrophic nitrification is always catalyzed by two different microorganisms. However, our knowledge of the survival strategies of comammox in complex ecosystems, such as full-scale wastewater treatment plants (WWTPs), remains limited. Analyses of genomes and *in situ* transcriptomes of four comammox organisms from two full-scale WWTPs revealed that comammox were active and showed a surprisingly high metabolic versatility. A gene cluster for the utilization of urea and a gene encoding cyanase suggest that comammox may use diverse organic nitrogen compounds in addition to free ammonia as the substrates. The comammox organisms also encoded the genomic potential for multiple alternative energy metabolisms, including respiration with hydrogen, formate, and sulfite as electron donors. Pathways for the biosynthesis and degradation of polyphosphate, glycogen, and polyhydroxyalkanoates as intracellular storage compounds likely help comammox survive unfavorable conditions and facilitate switches between lifestyles in fluctuating environments. One of the comammox strains acquired from the anaerobic tank encoded and transcribed genes involved in homoacetate fermentation or in the utilization of exogenous acetate, both pathways being unexpected in a nitrifying bacterium. Surprisingly, this strain also encoded a respiratory nitrate reductase which has not yet been found in any other *Nitrospira* genome and might confer a selective advantage to this strain over other *Nitrospira* strains in anoxic conditions.

## Abstract (precis)

The discovery of comammox in the genus *Nitrospira* changes our perception of nitrification. However, genomes of comammox organisms have not been acquired from full-scale WWTPs, and very little is known about their survival strategies and potential metabolisms in complex wastewater treatment systems. Here, four comammox metagenome-assembled genomes and metatranscriptomic data sets were retrieved from two full-scale WWTPs. Their impressive and—among nitrifiers—unsurpassed ecophysiological versatility could make comammox *Nitrospira* an interesting target for optimizing nitrification in current and future bioreactor configurations.

## INTRODUCTION

Aerobic chemolithoautotrophic nitrification, the biological oxidation of ammonia to nitrate, is a crucial process of the nitrogen cycle in natural and engineered systems. Throughout the last century, nitrification was considered to be performed by two different guilds of microorganisms in cooperation. The first step, ammonia oxidation to nitrite, is carried out by the ammonia-oxidizing microorganisms, ammonia-oxidizing bacteria (AOB) and archaea (AOA). The second step, nitrite oxidation to nitrate, is catalyzed by nitrite-oxidizing bacteria (NOB). The long-standing paradigm that this division of labor in nitrification would be obligate was questioned in a theoretical analysis (1) and finally refuted by the discovery of complete ammonia oxidizers (comammox organisms), members of the NOB-harboring genus *Nitrospira*, which catalyze both steps of nitrification on their own (2, 3). A subsequent physiological study (4) revealed a very high affinity for ammonia and a high specific growth yield of comammox *Nitrospira*, suggesting an oligotrophic lifestyle and yield-optimized survival strategy that is consistent with theoretical metabolic models of complete ammonia oxidation (1). Accordingly, comammox have been detected by metagenomics and PCR-based analyses in oligotrophic drinking water treatment systems, groundwater wells, and terrestrial subsurfaces (2, 5–9). Comammox *Nitrospira* have also been found in full-scale wastewater treatment plants (WWTPs) (2, 8, 10–12), but the extent of their contribution to nitrification in WWTPs remains to be determined.

Traditionally, *Nitrospira* were regarded as obligate chemolithoautotrophs that acquire energy for growth solely from nitrite oxidation. However, several *Nitrospira* are physiologically more versatile and can utilize various organic substrates in the presence of ammonia or nitrite (see, for example, references 13, 14, and 15). Moreover, the nitrite oxidizer Nitrospira moscoviensis can grow aerobically by hydrogen (H_2_) (16) and formate (14) oxidation in the absence of nitrite, and utilization of formate was also observed for other *Nitrospira* members (17, 18). Altogether, these findings demonstrated a much greater ecological flexibility of canonical nitrifiers than previously perceived. Therefore, in addition to analyses of comammox using markers, such as ammonia monooxygenase (*amoA*) genes (7, 8), whole-genome studies and gene expression or protein analyses are crucial to improving our understanding of comammox ecophysiology. Recently, the comammox organism *Nitrospira inopinata* was isolated, and the annotation of its genome revealed possible alternative lifestyles such as hydrogen and sulfide oxidation, the fermentation of carbohydrates, and dissimilatory nitrite reduction to ammonium (4). A metagenomic analysis of comammox in a nitrifying laboratory-scale reactor also identified H_2_ oxidation as a putative additional energy metabolism (19). However, in-depth genome- and gene expression-based analyses of comammox in full-scale WWTPs are still lacking. Here, four comammox *Nitrospira* genomes were recovered from metagenomic data sets of activated sludge from two full-scale WWTPs. The gene content of reconstructed genomes, combined with metatranscriptomic data, revealed a surprisingly high metabolic versatility of comammox *Nitrospira* in wastewater treatment systems.

## RESULTS AND DISCUSSION

### Recovery of comammox clade A *Nitrospira* MAGs from full-scale WWTPs.

**FIG 1:**
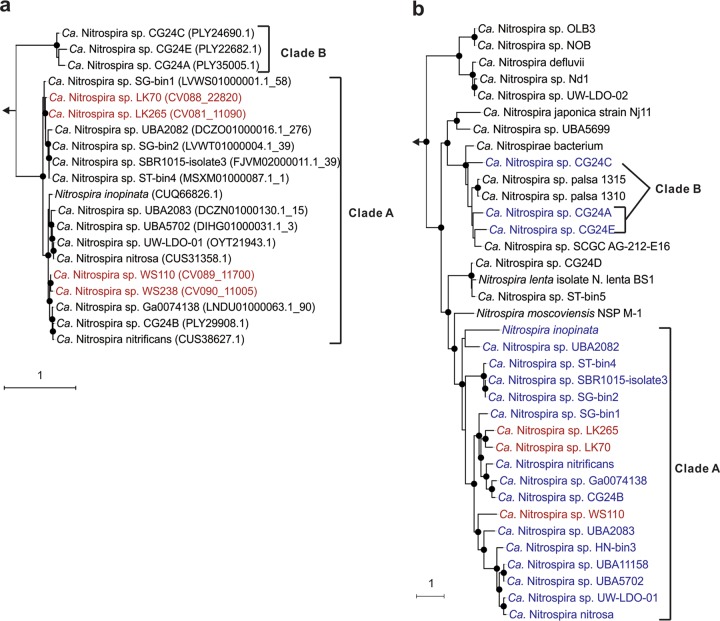
Phylogenetic analyses of comammox *Nitrospira*. (a) Maximum-likelihood tree of AmoA protein sequences showing the affiliation of the four comammox genomes acquired in this study (red) and previously published comammox genomes (black). The nodes with a bootstrap value of >85% are indicated as black solid dots. AmoA sequences of ammonia-oxidizing bacteria and archaea were used as outgroup. In all, 374 amino acid sequence alignment positions and 131 taxa (including outgroups) were considered. (b) Maximum-likelihood tree based on a concatenated sequence data set of 15 ribosomal proteins extracted from MAGs of LK70, LK265, and WS110 acquired in this study (red) (WS238 was excluded because of high contamination), previously published comammox genomes (blue), and genomes of nitrite-oxidizing *Nitrospira* (black). The nodes with a bootstrap value of >85% are indicated as black solid dots. Ribosomal proteins sequences of other members of the phylum *Nitrospirae* were used as outgroup. In total 48,088 amino acid sequence alignment positions and 69 taxa (including outgroups) were considered.

The AmoA sequences from the four new comammox metagenome-assembled genomes (MAGs)—Linkou 70 (LK70), LK265, Wenshan 110 (WS110), and WS238—clustered together with clade A comammox AmoA sequences from published fully or partially sequenced comammox genomes (Fig. 1a). Phylogenetic analyses of concatenated ribosomal protein (RP) sequences, which could be performed for MAGs LK70, LK265, and WS110, also confirmed the placement of the MAGs within comammox clade A (Fig. 1b). Close phylogenetic relationships between LK70 and LK265 recovered from plant LK, as well as between WS110 and WS238, recovered from plant WS were suggested by the AmoA, hydroxylamine oxidoreductase (HAO), and RP (only LK) phylogenies (Fig. S1a). In agreement with previous results (2, 3), comammox *Nitrospira* did not form a monophyletic group in an analysis based on the alpha subunit of nitrite oxidoreductase (NXR) (NxrA) from *Nitrospira* (Fig. S1b).

10.1128/mBio.03175-19.5 FIG S1 (a) Phylogenetic analysis of HAO sequences. Maximum-likelihood tree of HAO protein sequences showing the affiliation of HAO from the four comammox genomes acquired in this study (red) to HAO from previously published comammox genomes (blue). The nodes with a bootstrap value of >85% are marked in black solid dots. The tree was rooted with HAO sequences from canonical AOB and anammox organisms. In total, 1,152 amino acid sequence alignment positions and 45 taxa (including outgroups) were considered. (b) Phylogenetic analysis of NxrA sequences. Maximum-likelihood tree of NxrA protein sequences showing the affiliation of NxrA from comammox genomes acquired in this study (red) to NxrA from previously published comammox *Nitrospira* genomes (blue) and canonical nitrite-oxidizing *Nitrospira* (black). The short NxrA sequence in LK70 and three short NxrA sequences in WS238 were excluded. The nodes with a bootstrap value >85% were marked in black solid dots. The tree was rooted with NxrA sequences from anammox bacteria. In all, 1,151 amino acid sequence alignment positions and 93 taxa (including outgroups) were considered. (c) Phylogenetic analysis of [NiFe] hydrogenases. Maximum likelihood tree of [NiFe] hydrogenase protein sequences showing the affiliation of group 3b [NiFe] hydrogenases from comammox genomes acquired in this study (red) to [NiFe] hydrogenases from previously published comammox *Nitrospira* genomes (blue) and to reference sequences from the [NiFe] hydrogenase database HydDB (23) (black). Other [NiFe] hydrogenase sequences from HydDB were used as outgroup. The nodes with a bootstrap value of >85% are marked in black solid dots. In total 2,150 amino acid sequence alignment positions and 2,025 taxa were considered. Download FIG S1, JPG file, 0.6 MB. Copyright © 2020 Yang et al. 2020 Yang et al. This content is distributed under the terms of the Creative Commons Attribution 4.0 International license.

The four newly recovered comammox MAGs range in size from 2.4 to 4.5 Mb, with a completeness of 65 to 93% and a G+C content of 55.1 to 55.8% (Table S2). LK70 and WS110 are nearly complete MAGs with a low degree of contamination (Table S2). In comparison to 16 published comammox genomes (Table S2), the four MAGs had the highest ANI with “*Candidatus* Nitrospira nitrificans” (LK70, 80.9%; LK265, 80.3%; WS110, 76.2%; WS238, 75.3%) (Fig. S2). Consistent with the phylogenetic analyses, the MAGs from the same WWTP shared the highest ANI with each other (Fig. S2). However, their ANIs were still below the proposed species cutoff of 95% (20), suggesting that each MAG represents a novel comammox *Nitrospira* species. These four comammox strains have tentatively been named “*Ca*. Nitrospira sp. strain LK70,” “*Ca*. Nitrospira sp. LK265,” “*Ca*. Nitrospira sp. WS110,” and “*Ca*. Nitrospira sp. WS238.” Notably, WS238 was excluded from further analyses due to the high contamination level detected in this MAG.

10.1128/mBio.03175-19.2 TABLE S1 Quality parameters of the comammox *Nitrospira* genome LK70 after each round of iterative assembly. Abbreviations: Comp., completeness; Cont., contamination. Download Table S1, DOC file, 0.1 MB. Copyright © 2020 Yang et al. 2020 Yang et al. This content is distributed under the terms of the Creative Commons Attribution 4.0 International license.

10.1128/mBio.03175-19.3 TABLE S2 General features and sources of comammox *Nitrospira* genomes (completeness, >85%). Download Table S2, DOC file, 0.1 MB. Copyright © 2020 Yang et al. 2020 Yang et al. This content is distributed under the terms of the Creative Commons Attribution 4.0 International license.

10.1128/mBio.03175-19.6 FIG S2 Average nucleotide identity (ANI) analysis of comammox *Nitrospira* genomes. The heatmap illustrates the ANI-based similarities of published genomes of comammox organisms and the comammox genomes acquired in this study (names highlighted in red). Download FIG S2, JPG file, 0.3 MB. Copyright © 2020 Yang et al. 2020 Yang et al. This content is distributed under the terms of the Creative Commons Attribution 4.0 International license.

### Comammox *Nitrospira* are active in full-scale WWTPs.

Combined metagenomic and metatranscriptomic analyses provided the first holistic insights into the potential metabolic activities of comammox *Nitrospira* in full-scale WWTPs. To the best of our knowledge, this is the first *in situ* transcriptomic study of comammox in full-scale WWTPs. It serves as a source of hypotheses on the biology of comammox *Nitrospira*, and thus it provides a valuable starting point for follow-up research to explore how the genomic features and transcriptional activities discussed here are reflected by phenotypic traits of these mostly uncultured nitrifiers.

**FIG 2:**
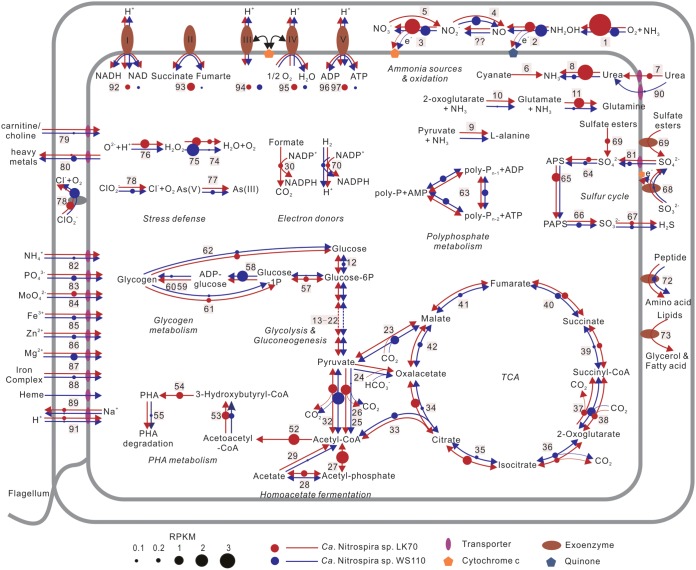
Cell metabolic cartoon constructed from the annotation of the nearly completely sequenced LK70 and WS110 comammox genomes and the metatranscriptomic data. Numbers at pathway steps match the numeric enzyme identifiers in Table S3. The diameters of circles represent the transcript abundances of the respective genes.

All four wastewater comammox strains transcribed *amo*, *hao*, and *nxr* genes (see the supplemental material), suggesting that they were actively oxidizing ammonia to nitrate. Transcripts of the respiratory chain complexes I, II, and III and the F-type ATP synthase were all detected in the two nearly complete MAGs LK70 and WS110 (Fig. 2; Table S3). Interestingly, in addition to the F-type ATP synthase, the two MAGs from plant LK (LK70 and LK265) also encode a V-type ATPase. To date, the occurrence of both an F-type and a V-type ATP synthase has been reported only for one other *Nitrospira* draft genome from a terrestrial subsurface sample (9). However, only the transcript of its subunit I was detected in strain LK70. A V-type ATPase in acid-tolerant AOA contributes to pH homeostasis (21), but its role in neutrophilic comammox organisms remains unknown. Like the other NOB and comammox *Nitrospira* (see, for example, references 4, 14, and 15), these comammox strains do not encode any canonical heme-copper oxidase. Instead, they code for and transcribed a novel cytochrome *bd*-like heme-copper oxidase (Fig. 2; Table S3) that is most likely complex IV of *Nitrospira* (4, 15).

10.1128/mBio.03175-19.4 TABLE S3 (a) Summary of functional genes of the four newly acquired comammox *Nitrospira* analyzed in this study. (b) Transcripts (RPKMs) of predicted genes of the four newly acquired comammox *Nitrospira* analyzed in this study. Download Table S3, XLSX file, 0.3 MB. Copyright © 2020 Yang et al. 2020 Yang et al. This content is distributed under the terms of the Creative Commons Attribution 4.0 International license.

### Nitrogen metabolism of comammox in WWTPs.

**FIG 3:**
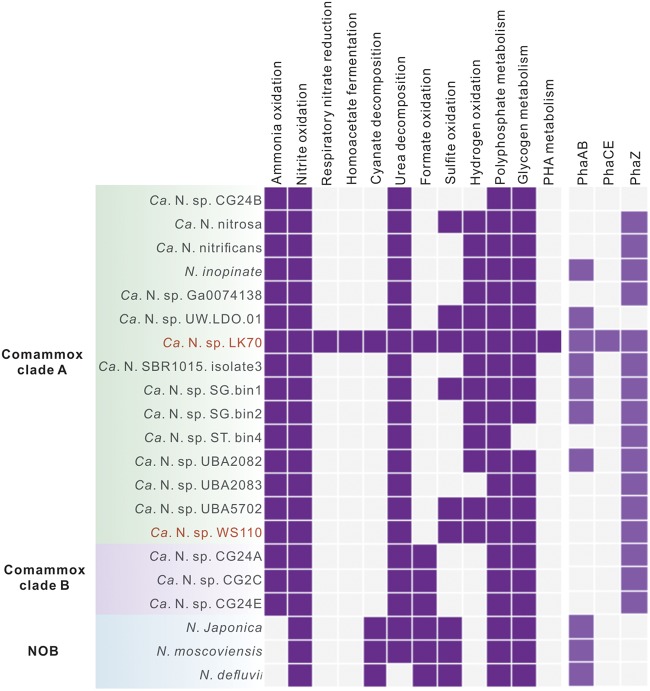
Distribution of key pathways, including nitrification, the use of organic nitrogen compounds, alternative energy metabolisms, and storage compound metabolisms, in the two almost completely reconstructed comammox genomes acquired in this study (names highlighted in red), previously published comammox *Nitrospira* genomes, and three completely sequenced genomes of canonical *Nitrospira* (NOB). Blue indicates the presence and gray indicates the absence of the respective pathway. PhaA, acetyl-CoA C-acyltransferase; PhaB, acetoacetyl-CoA reductase; PhaCE, class III poly(R)-hydroxyalkanoic acid synthase subunits C and E; PhaZ, poly(3-hydroxybutyrate) depolymerase.

As expected, the genes of the known key enzymes for ammonia oxidation (*amoABCDE* and *haoAB*-*cycAB*) and for nitrite oxidation (*nxrABC*) by comammox *Nitrospira* (2, 3) were identified (see the supplemental material and Table S3), and their transcripts were detected in the four comammox MAGs (Fig. S3a to d). Because the low completeness and relative high contamination of MAGs LK265 and WS238 (Table S2) could introduce biases in physiological interpretations, we now focused our analysis on the almost complete MAGs LK70 and WS110. Further information on the nitrogen metabolism-related genes in LK265 and WS238 can be found in Table S3. In addition to the aforementioned core genes of nitrification, the two almost complete MAGs, LK70 and WS110, code for urease (*ureABC*) with gene transcription (Fig. 2; Table S3). Although free ammonia is very likely the main substrate for nitrification in domestic WWTPs, the presence and transcription of urease genes are consistent with the possible availability of urea as an additional source of ammonia in wastewater and support the previous notion that urea may be utilized for energy conservation and nitrogen assimilation by comammox *Nitrospira* in WWTPs (3, 4, 19, 22). However, only LK70 encodes a known urea ATP-binding cassette transporter (*urtABCDE*) (Fig. 2). Interestingly, the gene of a putative short-chain amide porin that may be involved in exogenous urea acquisition (23) was found in LK70 and WS110 (Table S3) and was transcribed by WS110 (Fig. 2). Urease genes have also been found in other comammox genomes (Fig. 3) (4, 9, 22) including data sets from nitrifying bioreactors (3, 19), and urea cleavage has been observed for an enrichment culture of the comammox strains “*Ca*. Nitrospira nitrosa” and “*Ca*. Nitrospira nitrificans” (3).

10.1128/mBio.03175-19.7 FIG S3 Transcript abundance levels of comammox *Nitrospira* strain LK70 (a), LK265 (b), WS110 (c), and WS238 (d). Colored arrows and labels highlight key genes of ammonia and nitrite oxidation, as well as genes for the use of organic nitrogen compounds. Ure, urease. Download FIG S3, JPG file, 0.7 MB. Copyright © 2020 Yang et al. 2020 Yang et al. This content is distributed under the terms of the Creative Commons Attribution 4.0 International license.

**FIG 4:**
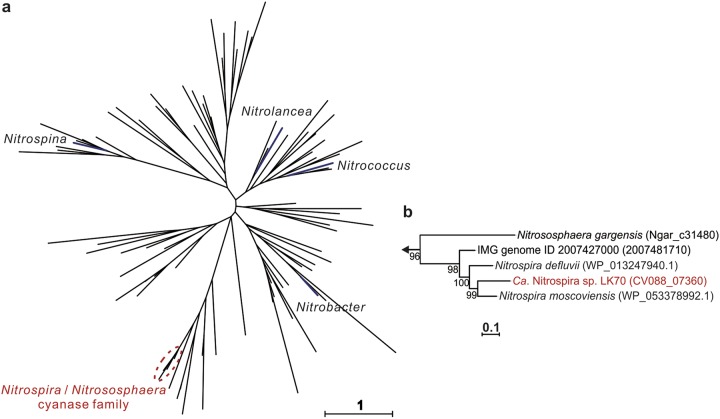
Phylogenetic analysis of cyanase sequences. (a) Unrooted maximum-likelihood tree highlighting the lineages that contain cyanases from canonical NOB (blue). Note that the *Nitrospira* lineage also contains the cyanase of the AOA *Nitrososphaera gargensis* (24). Branches including only cyanases from nonnitrifiers are not labeled. (b) Expanded view showing the placement of the comammox *Nitrospira* cyanase from LK70 (red) within the *Nitrospira*/*Nitrososphaera* cyanase family. In all, 340 amino acid sequence alignment positions and 93 taxa were considered.

Recent studies demonstrated the utilization of cyanate as a substrate for ammonia oxidation and nitrogen assimilation by thermophilic and marine AOA and marine anammox organisms (24–26). In the AOA strain *Nitrososphaera gargensis*, this capability is based on the release of ammonia from cyanate by the enzymatic activity of cyanate hydratase (cyanase) (24). Cyanase genes commonly occur in canonical NOB, including strictly nitrite-oxidizing *Nitrospira* members (Fig. 3). However, cyanase has so far been identified in only one canonical ammonia oxidizer, *N. gargensis* (24), and has only recently been found in two comammox MAGs (https://www.biorxiv.org/content/10.1101/529826v4) (Fig. 3). Intriguingly, in the comammox MAG LK70, we identified a gene encoding cyanase (*cynS*) (Table S3; Fig. 2). The presence of *cynS* in this comammox genome was confirmed by rigorous, iterative reassembly of the MAG (see the supplemental material and Fig. S6). According to a BLASTP search of the NCBI nr database, LK70 cyanase has the highest amino acid identity (78.77%) to the cyanase of the NOB Nitrospira moscoviensis. The close affiliation of the LK70 cyanase with homologs from nitrite-oxidizing *Nitrospira* was confirmed by a phylogenetic analysis (Fig. 4). Although *in situ* transcription of *cynS* by LK70 was not detected, the cyanase could enable this comammox strain to use cyanate as a substrate in WWTPs or other environments. Since abiotic urea degradation can lead to cyanate formation (27), the utilization of cyanate as an energy source may be an ecological advantage in urea-containing wastewaters and could be a distinguishing feature of strain LK70 compared to other comammox *Nitrospira* and canonical ammonia oxidizers. However, a recent study revealed cyanate oxidation to nitrite by marine AOA that lack canonical cyanase genes, indicating the possible existence of another, yet unidentified biochemical pathway for cyanate utilization (26). Thus, we cannot exclude the possibility that cyanate degradation may also occur in comammox organisms lacking any currently identifiable cyanases.

Despite living in a nonaerated tank, strain LK70 transcribed its nitrification genes. We can exclude the possibility of residual transcripts from the aerobic stage, because at WWTP LK all aerobic tanks are downstream of the anaerobic stage, and no activated sludge is returned from the aerobic tanks into the anaerobic tank (28). Instead, we assume that parts of the LK70 population had access to residual dissolved oxygen (Table 1), for example, at the outermost shell of activated sludge flocs. In contrast, LK70 cells located in the inner zones of flocs would rather be expected to use anaerobic energy metabolisms (see below). However, since the metatranscriptomic data set did not allow us to distinguish the activities in different microniches, such a spatial-functional segregation could not be verified in this study.

**TABLE 1:** Key parameters of the two WWTPs that were the source of the comammox genomes and metagenomes analyzed in this study^*a*^

Plant	Sampling site	Sampling site description			NH_4_^+^ (mg/liter)		NO_3_^−^ (mg/liter)		COD (mg/liter)		Reactor operation					DO (Inf, mg/liter)
		DO (mg/liter)	pH	*T* (°C)	Inf	Eff	Inf	Eff	Inf	Eff	Strategy	Influent (m^3^/day)	ORP (mV)	HRT (day)	Sampling reactor aeration (DO, mg/liter)	
Linkou (LK)	Anaerobic tank	0.02	7.4	25.3	63 ± 17	1 ± 0	0	24±10	13,439 ± 9,276^*b*^	11,206 ± 3,396	AOAOA	8,070	−80	14	0	2.09 ± 0.46
Wenshan (WS)	Deep oxidation ditch	0.07	6.7	21.2	118 ± 15	6 ± 2	11 ± 8	67 ± 15	389 ± 40	199 ± 54	Aeration	158.3	215	1	∼0.3 (night) ∼1.0 (day)	NA

a The indicated DO, pH, and temperature values were measured in the activated sludge samples taken for this study. NH_4_^+^, NO_3_^–^, and COD concentrations are average values from a 1-month period before the time of sampling. Abbreviations: AOAOA, anaerobic-aerobic-anoxic-aerobic-anoxic; COD, chemical oxygen demand; HRT, hydraulic retention time; NA, not applicable; ORP, oxidation-reduction potential; *T*, temperature; Inf, influent wastewater; Eff, effluent wastewater; DO, dissolved oxygen.

b The influent wastewater contains supernatant and filtrate from sludge thickening and dewatering, respectively.

### Autotrophy and storage compounds.

Since chemolithoautotrophic organisms, comammox, and canonical *Nitrospira* use CO_2_ as their carbon source through the reductive tricarboxylic acid (rTCA) cycle (2–4, 15). Pyruvate:ferredoxin oxidoreductase (POR), 2-oxoglutarate:ferredoxin oxidoreductase (OGOR), ATP-citrate lyase (ACLY), and fumarate reductase (FRD) are the key enzymes of the rTCA cycle (29–31). All of them are encoded by the two nearly complete comammox MAGs LK70 and WS110 (Table S3), and their genes were transcribed *in situ* (Fig. 2).

Both LK70 and WS110 encode the gluconeogenesis and glycolysis (Embden-Meyerhof-Parnas) pathways, as well as the biosynthesis and degradation of glycogen, which were also expressed (Table S3; Fig. 2). These pathways are also present in other comammox and canonical nitrite-oxidizing *Nitrospira* strains (2, 14, 15, 32) (Fig. 3). Storage of carbon and energy in the form of glycogen should help comammox organisms and NOB cope with fluctuations in substrate availability. Such shifts are probably common in many natural habitats and occur also in WWTPs, for example, when the concentrations of ammonium, nitrite, and dissolved oxygen change regularly in nitrifying and denitrifying bioreactors.

In addition to glycogen as a storage compound, LK70 encodes a potential biosynthesis pathway for polyhydroxyalkanoates (PHA). Genes of acetyl coenzyme A (acetyl-CoA) C-acyltransferase (*phaA*), acetoacetyl-CoA reductase (*phaB*), and class III poly(R)-hydroxyalkanoic acid synthase subunits C and E (*phaCE*) were identified (Table S3) and are colocalized (Fig. S4). Consistently, LK70 also encodes a potential poly(3-hydroxybutyrate) depolymerase (*phaZ*) that is involved in PHA degradation (33, 34). PHAs are usually formed under conditions of carbon excess and nitrogen or phosphate limitation (35, 36) as carbon and energy storage compounds (37–39). The terminal step of PHA synthesis is catalyzed by PhaCE (33, 40). Interestingly, no homolog of *phaCE* has been identified before in other *Nitrospira* (comammox and NOB) genomes, although some of these *Nitrospira* genomes contain putative *phaAB* and *phaZ* genes (Fig. 3). Therefore, the presence of *phaZ* in other comammox genomes in the absence of a complete set of known PHA biosynthesis genes has been discussed as a possible relic (22). Since homologs of *phaZ* were transcribed by WS110 that does not contain *phaCE* either (Table S3), the function of *phaZ* in comammox *Nitrospira* deserves further investigation. However, the genetic inventory of LK70 for both PHA synthesis and degradation suggests that at least some comammox strains gain additional physiological flexibility by forming PHA. Transcription of the complete PHA biosynthesis pathway in LK70 (Fig. 2) indicates the potential relevance of PHA formation under the microoxic conditions in the nonaerated tank (Table 1), which was the source of LK70. It is tempting to speculate that a fraction of the acetyl-CoA formed in the course of anaerobic metabolism (see below), or exogenous organics taken up from the sludge liquor in WWTP LK that was high in COD (Table 1), could be stored as PHA.

10.1128/mBio.03175-19.8 FIG S4 Schematic illustration of genomic loci encoding class I, II, and III PHA synthases in the comammox organisms LK70 and LK265 and in reference genomes of other bacteria. Genes are drawn to scale. Download FIG S4, JPG file, 0.2 MB. Copyright © 2020 Yang et al. 2020 Yang et al. This content is distributed under the terms of the Creative Commons Attribution 4.0 International license.

Both LK70 and WS110 encode polyphosphate kinases, which have also been identified in other genomes of comammox and canonical *Nitrospira* (see, for example, references 2 and 15) (Fig. 3). Thus, *Nitrospira* seems to commonly use polyphosphate for the intracellular storage of phophorus and energy. Polyphosphate kinases in the PPK1 and PPK2 families preferentially catalyze the polymerization and degradation of polyphosphate, respectively (41, 42). Strain WS110 transcribed one of its *ppk2* genes (Fig. 2).

### Utilization of organic substrates and fermentation.

Organic compounds, such as glycogen and PHA, may be degraded by LK70 and WS110 *via* the canonical oxidative tricarboxylic acid (oTCA) cycle. The respective genes were identified in both MAGs, except for 2-oxoglutarate dehydrogenase, which was found in LK70 but not in the recovered parts of the WS110 genome (Table S3). Most genes of the oTCA cycle (the majority of which are shared with the rTCA cycle) were transcribed in both strains (Fig. 2). This included a hallmark enzyme of the oTCA cycle, 2-oxoglutarate dehydrogenase, in LK70 (Fig. 2). Operation of the oTCA cycle in LK70 living in a nonaerated tank would make sense in the context of respiration of organic substrates with nitrate as the terminal electron acceptor. This could be possible if, like in canonical *Nitrospira* (14, 32), the periplasmic NXR of LK70 is a reversible enzyme and also capable of nitrate reduction to nitrite. Moreover, all genes of a membrane-associated and cytoplasmically oriented respiratory nitrate reductase, NAR (*narGHIJ*), were identified in LK70 (Fig. 2; Table S3). This finding was unexpected, because other *Nitrospira* strains use their periplasmic NXR for catabolic nitrate reduction (see above) and NAR has not yet been found in any other *Nitrospira* genome (Fig. 3). Iterative reassembly of the LK70 MAG did not contradict the presence of the *nar* genes in LK70 (Fig. S6). Nevertheless, this result should be confirmed by resequencing of the genome and physiological experiments, once an enrichment culture or isolate of this organism has been obtained. In our study, *in situ* transcription of the *narI* gene encoding the membrane-integral gamma subunit of NAR was detected. From a bioenergetic perspective, the cytoplasmically oriented NAR could be a more efficient nitrate reductase than a periplasmic enzyme (43). Hence, in anoxic conditions NAR might confer a selective advantage to nitrate-reducing LK70 over other *Nitrospira* strains that possess only NXR. However, comparisons are difficult as long as only little is known about the periplasmic NXR of *Nitrospira* with regard to its exact subunit composition, bioenergetic properties, and interactions with other protein complexes in the electron transport chain (15).

Interestingly, according to its genetic inventory (Table S3), LK70 might be capable of homoacetate fermentation for chemoorganotrophic energy conservation under anoxic conditions. In this case, acetyl-CoA, carbon dioxide, and reduced ferredoxin could be produced from pyruvate by POR acting in the reverse direction to that used for CO_2_ fixation (Fig. 2). Subsequently, acetyl-CoA would be converted to acetyl phosphate by phosphate acetyltransferase and further to acetate, with ATP production, by acetate kinase (Fig. 2). Notably, it remains unclear how the electrons, which are transferred from pyruvate to ferredoxin in the POR reaction, are dissipated. This could theoretically be accomplished by a H_2_-evolving hydrogenase. Coupling of homoacetate fermentation with H_2_ evolution has already been proposed for other organisms (44–46). However, no hydrogenase known to form H_2_ with electrons from ferredoxin was identified in the sequenced part of the LK70 genome.

Alternatively, acetate kinase and phosphate acetyltransferase might both operate in the reverse direction to that used for fermentation and catalyze the synthesis of acetyl-CoA from acetate (Fig. 2). The acetyl-CoA could then serve as a substrate for the oTCA cycle and respiration or for PHA biosynthesis (Fig. 2). Acetyl-CoA could also be converted to pyruvate by POR (Fig. 2), thus saving LK70 some of the energy needed for the *de novo* biosynthesis of pyruvate by CO_2_ fixation (Fig. 2). Hence, it seems that strain LK70 might also be able to use exogenous acetate as a source of energy and/or carbon.

The genes of acetate kinase and phosphate acetyltransferase were transcribed *in situ* by LK70 (Fig. 2), suggesting that acetate metabolism was active in this organism. It remains to be determined whether LK70 uses homoacetate fermentation to degrade intracellular glycogen or exogenous organic substrates in the nonaerated tank, or whether LK70 takes up and utilizes acetate that may be produced by other organisms under the oxygen-deprived conditions in WWTP LK (Table 1).

### Alternative electron donors.

Both MAGs LK70 and WS110 contain all genes of a group 3b [NiFe] hydrogenase and the factors required for hydrogenase maturation (Table S3). Group 3b hydrogenases are widely distributed among phylogenetically diverse bacteria and archaea (47). Their genes have also been reported in the genomes of comammox *Nitrospira* (3, 4, 9) and the marine, canonical NOB Nitrospina gracilis (48). Group 3b hydrogenase genes commonly occur in clade A comammox genomes (Fig. 3; Fig. S1c). These hydrogenases might couple NAD(P)H oxidation to the evolution of H_2_ (49). At ∼20°C this reaction would be highly inefficient and could proceed only at a low partial pressure of H_2_ around 10 to 100 Pa (50), thus precluding a role of the 3b hydrogenase in homoacetate fermentation (see above). However, 3b hydrogenases may also be reversible, oxidizing H_2_ with NAD(P)^+^ as electron acceptor (47, 49, 51). At least some 3b hydrogenases also act as sulfhydrogenases that transfer electrons from NAD(P)H to elemental sulfur or polysulfide and thus produce H_2_S (47, 51). In comammox *Nitrospira*, group 3b hydrogenases may be involved in energy conservation by aerobic H_2_ oxidation, a lifestyle already demonstrated for the NOB *N. moscoviensis* based on the activity of a group 2a hydrogenase (16). The detected transcription of the group 3b hydrogenase by LK70 and WS110 (Fig. 2) indicates that hydrogen metabolism could be important for comammox *Nitrospira* in WWTPs.

Formate can be used as a carbon and also as an energy source by the NOB *N. moscoviensis* (14) and *Nitrospira japonica* (18), and uptake of ^14^C from labeled formate was observed for uncultured *Nitrospira* in activated sludge (17). Formate dehydrogenase genes have been identified in the genomes of *N. moscoviensis* (14), clade B comammox (22), and a recently published clade A comammox draft genome designated as “*Ca*. Nitrospira sp. strain RCA” (9), but not yet in other known clade A comammox organisms (Fig. 3). The here recovered clade A comammox strain LK70 encodes genes of a molybdenum-dependent formate dehydrogenase (*fdhF*) and an accessory sulfurtransferase (*fdhD*) that may enable LK70 to utilize formate. In the nonaerated tank at WWTP LK, H_2_ and formate could be released by other fermenting organisms. These substrates would then be available for aerobic respiration by LK70 cells that have access to dissolved oxygen, for example if they grow in the outer shell of activated sludge flocs, or for nitrate reduction as observed already for *N. moscoviensis* (14, 32).

Both LK70 and WS110 encode a periplasmic sulfite dehydrogenase (Fig. 2; Table S3), which could couple sulfite oxidation to sulfate with the reduction of cytochrome *c* as suggested for *N. gracilis* (48). Transcripts of the sulfite dehydrogenase genes were detected for strain WS110 (Fig. 2). Genes of sulfite dehydrogenase have also been identified in some other clade A comammox genomes and the closed genomes of three nitrite-oxidizing *Nitrospira* (Fig. 3).

### Stress defense.

Both comammox genomes LK70 and WS110 contain genes coding for superoxide dismutase (SOD), catalase, and several peroxidases (Fig. 2; Table S3) and thus are well prepared for defense against reactive oxygen species (ROS). Except for SOD encoding gene in WS110, transcripts of all ROS defense genes were detected (Fig. 2). This is remarkable, since many *Nitrospira* lack a complete set of ROS detoxification enzymes. For example, the comammox strains *N. inopinata* and “*Ca.* Nitrospira nitrosa” do not encode SOD, and the NOB *N. defluvii* does not possess SOD or catalase (2, 3, 15). Considering that *N. defluvii* is also a wastewater organism (15, 52), it seems that *Nitrospira* in WWTPs use different and partly unknown pathways to detoxify ROS.

The LK70 and WS110 genomes also encode several other mechanisms for dealing with environmental stress (Fig. 2; Table S3): a glycine betaine/carnitine/choline transport system, which could contribute to osmoregulation and temperature adaptation by transporting compatible solutes into the cells (53, 54); a CusA/CzcA family heavy metal efflux RND transporter, which may increase the resistance to elevated heavy metal concentrations in sewage (55); and chlorite dismutase (CLD)-like enzymes that also occur in other *Nitrospira* strains and could detoxify chlorite (56, 57). The substrate of CLD might be chlorite, which is produced during the reduction of chlorate by NOB (58), or an unknown compound. However, bacterial CLD-like enzymes are phylogenetically, structurally, and functionally diverse (59), and the primary physiological role of CLD in *Nitrospira* and other NOB (60) is unknown. In addition, both LK70 and WS110 possess flagella and chemotaxis genes, which should enable them to find favorable microhabitats within the complex structure of activated sludge flocs and biofilms, and LK70 contains a regularly interspaced short palindromic repeats (CRISPR) system for phage defense (61) (Table S3).

### Coexistence of nitrifying microorganisms in the studied WWTPs.

The two WWTPs, LK and WS, also harbored canonical nitrifiers in addition to the comammox organisms. The transcriptional activities of these canonical nitrifiers, comammox, and also anammox and denitrifiers have been compared in a previous study (28) and are summarized in Fig. S5.

10.1128/mBio.03175-19.9 FIG S5 Comparison of the abundances and total gene transcripts of comammox *Nitrospira* and canonical nitrifiers based on the metagenomic and metatranscriptomic datasets, respectively, retrieved from the WWTPs LK (a) and WS (b). Transcript abundances of *amoA* and *nxrA* genes recovered from the assembled MAGs of comammox *Nitrospira* and canonical nitrifiers retrieved from the WWTPs LK (c) and WS (d) are also shown. Download FIG S5, TIF file, 0.1 MB. Copyright © 2020 Yang et al. 2020 Yang et al. This content is distributed under the terms of the Creative Commons Attribution 4.0 International license.

10.1128/mBio.03175-19.10 FIG S6 Sequencing coverage of selected key genes of comammox strain LK70 after 60 rounds of iterative genome assembly, including ammonia monooxygenase (*amoCBA*) (a), cyanase (*cynS*) (b), phosphate acetyltransferase (*pat*) (c), formate dehydrogenase (*fdhF*), nitrate reductase (*narIJHG*) (d), class III poly(R)-hydroxyalkanoic acid synthase subunit PhaC (*phaCE*) (e), and poly(3-hydroxybutyrate) depolymerase-like protein (*pdl*) (f). (g) A heatmap illustrates consistency in tetranucleotide composition of the comammox LK70 genome after 60 rounds of iterative assembly. Rows represent contigs, and columns represent unique tetranucleotides. The intensity of the heatmap is colored according to log-transformed tetranucleotide composition of each contig, normalized to the contig length. Contigs containing highlighted genes in panels a to f are indicated in color and labeled in the left column. (h to i) Consistency in coverage between contigs of the comammox LK70 genome after 60 rounds of iterative assembly. (h) A histogram of the contig median coverage (i) Comparisons of the gene contents between LK70 and the reassembled LK70.round.60. Download FIG S6, JPG file, 0.3 MB. Copyright © 2020 Yang et al. 2020 Yang et al. This content is distributed under the terms of the Creative Commons Attribution 4.0 International license.

In WWTP LK, comammox coexisted with canonical AOB (Fig. S5a). In the metagenome from this system, neither AOA nor strictly nitrite-oxidizing *Nitrospira* or any other canonical NOB were detected and comammox was by far the most abundant and active known nitrifier (Fig. S5a). In contrast, comammox cooccurred with AOA, AOB, and NOB (the latter also from the genus *Nitrospira*) in WWTP WS. The comammox strains were less abundant than the canonical nitrifiers (Fig. S5b). Moreover, plant WS also contained anammox organisms (data not shown) that likely competed with the aerobic nitrifiers for ammonium and nitrite. Notably, no anammox organisms were detected in the metagenome from WWTP LK although this tank was not aerated. The different nitrifier community compositions in the two WWTPs at the metagenomic level were consistent with the abundances of different *amoA* and *nxrA* transcripts. These data indicate that comammox *Nitrospira* could be the functionally predominant nitrifiers in plant LK, whereas canonical nitrifiers likely are more important in plant WS (Fig. S5c, d). These results are in agreement with previous findings that the distribution of comammox *Nitrospira* in full-scale WWTPs is highly variable (2, 8, 10–12). However, the abundance of an organism does not always reflect its contribution to an environmental process, such as nitrification (62). Thus, follow-up research that quantifies the actual contributions of comammox and canonical nitrifiers to nitrification in different WWTPs and natural habitats, taking into account the impact of fluctuating environmental conditions and alternative energy metabolisms, is urgently needed. Metagenomic and gene expression analyses, such as our study, prepare this next step and provide a knowledge basis by identifying the potential functional key players and their potential metabolic pathways and alternative lifestyles.

### Conclusions.

The metagenomic reconstruction of four comammox MAGs derived from two full-scale WWTPs, combined with a metatranscriptomic analysis, has revealed a substantial and previously unknown potential metabolic versatility of comammox *Nitrospira* in wastewater. At least some comammox organisms can apparently utilize not only free ammonia but also urea and cyanate as substrates for chemolithoautotrophic complete nitrification. In particular, the discovery of a cyanase gene in a comammox genome is a remarkable addition to previous knowledge that only certain AOA and marine anammox organisms are able to cleave cyanate for ammonia oxidation. Moreover, comammox *Nitrospira* in WWTPs seem to be highly flexible with regard to alternative energy metabolisms. Unexpectedly, their inventory of such pathways may include an anaerobic lifestyle based on the fermentation of organic compounds. Interestingly, a putative pathway for the facilitated fermentation of aromatic amino acids coupled to H_2_ release has recently been found in the genome of a thermophilic canonical AOA strain (63). These and our results indicate the possible presence of fermentative pathways in phylogenetically and ecologically diverse nitrifiers. It remains to be shown whether these organisms can anaerobically grow by fermentation or rely on these pathways only to persist during periods of limited oxygen and nitrate availability. In addition, comammox strains in WWTPs might be capable of reducing nitrate to nitrite with electrons from low-potential donors. This activity was already observed for canonical *Nitrospira* (14, 32). The utilization of nitrate as an alternative terminal electron acceptor would further increase the ecophysiological flexibility of comammox in alternately nitrifying and denitrifying bioreactors. The broad range of potential energy metabolisms is complemented by several pathways to make and consume intracellular storage compounds. This enables comammox cells to store energy and carbon, which could be used to express new enzymes and switch between different lifestyles when the environmental conditions change. In summary, comammox *Nitrospira* in WWTPs appear to be very well adapted to the complex wastewater environment, which is characterized by a plethora of (sometimes harmful) organic and inorganic substances, a large diversity of microhabitats within sludge flocs and biofilms, and frequent changes of key environmental parameters (e.g., the ammonium load or the dissolved oxygen concentration) during bioreactor operation. Future research efforts might aim to exploit the unique physiological versatility of comammox, which is unmatched by the known canonical nitrifiers, to optimize nitrogen removal from sewage in current and new bioreactor and process designs.

## MATERIALS AND METHODS

### Sample collection, sequencing, and data analysis.

Activated sludge was collected from the mainstreams of two full-scale WWTPs from Taiwan, including an anaerobic tank of the Linkou (LK) WWTP in New Taipei and a nitrifying deep oxidation ditch of the Wenshan (WS) WWTP in Taichung (Table 1) (28). A detailed description, including process flow diagrams, of the two WWTPs is provided in our previous study (28). Briefly, plant WS is equipped with a deep oxidation ditch for biological nitrogen removal; the aeration in plant LK is configured as an anaerobic-aerobic-anoxic-aerobic-anoxic (AOAOA) system (Table 1). Activated sludge from the anaerobic tank of plant LK was sampled because the ammonia added was fully removed by this tank (Table 1). Supernatant from sludge thickening and filtrate from dewatering were mixed with the influent of the anaerobic tank. The influent wastewater of plant WS is a mixture of car-washing wastewater, landfill leachate, and supernatant of kitchen waste compost, whereas the influent of plant LK is municipal wastewater (Table 1). The chemical oxygen demand (COD) of the influent wastewater of plant LK was extremely high because of the inputs of supernatant and filtrate from sludge thickening and dewatering (Table 1).

Three independent activated sludge samples (technical replicates) were collected from each tank, and samples for DNA isolation were stored at –20°C, while samples for RNA isolation were preserved on site in LifeGuard soil preservation solution (Qiagen, Germany). Details of sampling, nucleic acid extraction, cDNA synthesis, and sequencing, as well as *de novo* metagenomic assembly, binning, and quality assessment, have been described in a previous study (28). Briefly, total DNA and RNA were extracted from each replicate sample, and the DNA or RNA, respectively, extracted from each sample was pooled. RNA was converted to double-stranded cDNA. The acquired DNA and cDNA were used for metagenomic and metatranscriptomic sequencing, respectively. The trimmed metagenomic data sets were assembled *de novo* using IDBA-UD v1.1.1 (64) using the parameters -mink 65, -maxk 145, and -step 8, and resulting scaffolds were binned using Maxbin with the setting “-min_contig_length 2500” (65). The rRNA reads were identified and removed from metatranscriptomic data sets using RiboPicker (66).

Finally, four comammox metagenome-assembled genomes (MAGs) were obtained; these were named LK70, LK265, WS110, and WS238. The completeness and level of contamination of the acquired draft genomes were estimated using CheckM v1.0.6 (67). In order to confirm that the novel genes identified in LK70 (see below) were not contaminations in binning, this MAG was subjected to highly iterated and rigorous reassembly (see the supplemental material and Fig. S6). Short metagenomic reads were mapped to the four MAGs by Bowtie2 v2.2.9 (68) with the defult settings to calculated the abundances of genomes as reads per kilobase per million (RPKM) (69). Non-rRNA metatranscriptomic reads were mapped to the predicted genes by BWA v0.7.17 (70) with the defult settings to calculate the transcripts of genes as RPKM as follows: RPKM = (number of mapped reads)/[(gene length/1,000) × (total mapped reads/1,000,000)].

### Phylogenetic analyses.

A previously reported syntenic block of 15 universal ribosomal proteins (RP: L2, 3, 4, 5, 6, 14, 15, 18, 22, and 24; S3, 8, 10, 17, and 19) (71) was extracted from the new comammox *Nitrospira* MAGs acquired in this study, previously published comammox genomes before April 2019 (completeness > 85%) (Table S2), and additionally selected *Nitrospirae* genomes. Each set of RP amino acid sequences was aligned using MAFFT (72), and individual RP alignments were concatenated with an in-house R script and trimmed with trimAl with the setting “-gt 0.1” (73). Because of high contamination, MAG WS238 was excluded from the RP phylogenetic analysis. Prior to phylogenetic analyses, the respective protein sequences of AmoA, HaoA, and NxrA from previously published comammox genomes were used to generate reference databases. Following open reading frame (ORF) prediction using Prodigal v2.6.3 (74), the homologous protein sequences in the reconstructed comammox MAGs were recovered by BLASTP searches against the respective reference databases using an E value threshold of <10^−10^. The blast results were filtered using a minimum sequence identity of 40% and minimum alignment length (length of aligned query sequence/length of database sequence) of 50%. The filtered sequences were then added to the respective databases. AmoA sequences from AOA and AOB were manually added to the AmoA database; HaoA sequences from AOB and anaerobic ammonium oxidizers (anammox bacteria) were manually added to the HaoA database. Phylogenetic analyses of NxrA comprised NxrA sequences from the genus *Nitrospira* and three of the recovered *Nitrospira* MAGs here, while the short NxrA sequences in LK70 and WS238 were excluded. The amino acid sequences of AmoA, HaoA, and NxrA were aligned with MAFFT (72), and the multiple sequence alignments were trimmed using trimAl with the setting “-gt 0.1” (73). Maximum-likelihood trees for functional gene alignments and the concatenated RP alignment were calculated using IQ-TREE with the default settings (75). The models of sequence evolution LG+R3, LG+R6, LG+R3, and LG+F+R10 were chosen from 546 protein sequence evolution models by ModelFinder (as implemented in IQ-TREE) to build AmoA, HaoA, NxrA, and RP phylogenetic trees, respectively.

To classify the [NiFe] hydrogenases encoded by comammox genomes, predicted ORFs were compared to sequences of the large subunit of the [NiFe] hydrogenases that were downloaded from HydDB (76) by BLASTP using an E value cutoff of <10^−10^. The blast results were filtered as described above, and the filtered sequences were submitted to the HydDB online classifier for hydrogenases (https://services.birc.au.dk/hyddb/) (76). A maximum-likelihood phylogenetic tree of large subunit [NiFe] hydrogenase sequences identified in comammox genomes and of reference sequences from HydDB was constructed as described above using the model LG+R3. A maximum-likelihood phylogenetic tree of cyanase coding genes, including the cyanase from MAG LK70 and a previously reported 99 representative cyanase coding gene data set (24), was constructed as described above using the model WAG+R5. Phylogenetic trees were visualized using iTOL (77).

### Genome analyses.

MAGs were annotated by GhostKOALA, KEGG’s internal annotation tool for the K number assignment of KEGG GENES using the SSEARCH computation (78). In addition, predicted ORFs were assigned to existing clusters of orthologous groups (COGs) by eggNOG-mapper (79). ORFs were also analyzed by BLASTP searches against the NCBI nr database using an E value of <10^−5^ as a threshold with the setting “-max_target_seqs 3.” The blast hits for selected ORFs with interesting putative functions were compared to the KEGG and eggnog annotation results. Inconsistent results were further inspected by BLASTP searches against the Reference Proteins and UniProtKB/Swiss-Prot databases with an E value threshold of <10^−10^ and/or by phylogenetic analysis with reference sequences. To identify potentially secreted proteins, ORFs were screened for signal peptides using SignalP 4.1, Signal-BLAST, and PSORTb (80–82). The gene annotations of the four comammox MAGs are summarized in Table S3.

Pairwise average nucleotide identity (ANI) was calculated between the four comammox MAGs from this study, previously published comammox genomes (completeness > 85%) (Table S2), and four closed NOB *Nitrospira* genomes using OrthoANI (83).

### Data availability.

Raw metagenomic and metatranscriptomic sequences have been submitted to NCBI under BioProject PRJNA406858. The comammox MAGs (LK70, LK265, WS110, and WS238) are available in NCBI under accession numbers SAMN07644402, SAMN07644401, SAMN07644400, and SAMN07644399. The four MAGs are also available in eLMSG (an eLibrary of Microbial Systematics and Genomics, https://www.biosino.org/elmsg/index) under accession numbers LMSG_G000000182.1, LMSG_G000000183.1, LMSG_G000000184.1, and LMSG_G000000185.1.

10.1128/mBio.03175-19.1 TEXT S1 Supplemental notes regarding core genes of complete ammonia oxidation. Supporting information is also included, as well as novel comammox MAG LK70 reassembly and references. Download Text S1, DOCX file, 0.05 MB. Copyright © 2020 Yang et al. 2020 Yang et al. This content is distributed under the terms of the Creative Commons Attribution 4.0 International license.
