# Supplementary figures and images for: Activity and Metabolic Versatility of Complete Ammonia Oxidizers in Full-Scale Wastewater Treatment Systems
Source: mBio. 2020 Mar 17;11(2):e03175-19. doi: 10.1128/mBio.03175-19 (PMC7078480; doi:10.1128/mBio.03175-19)

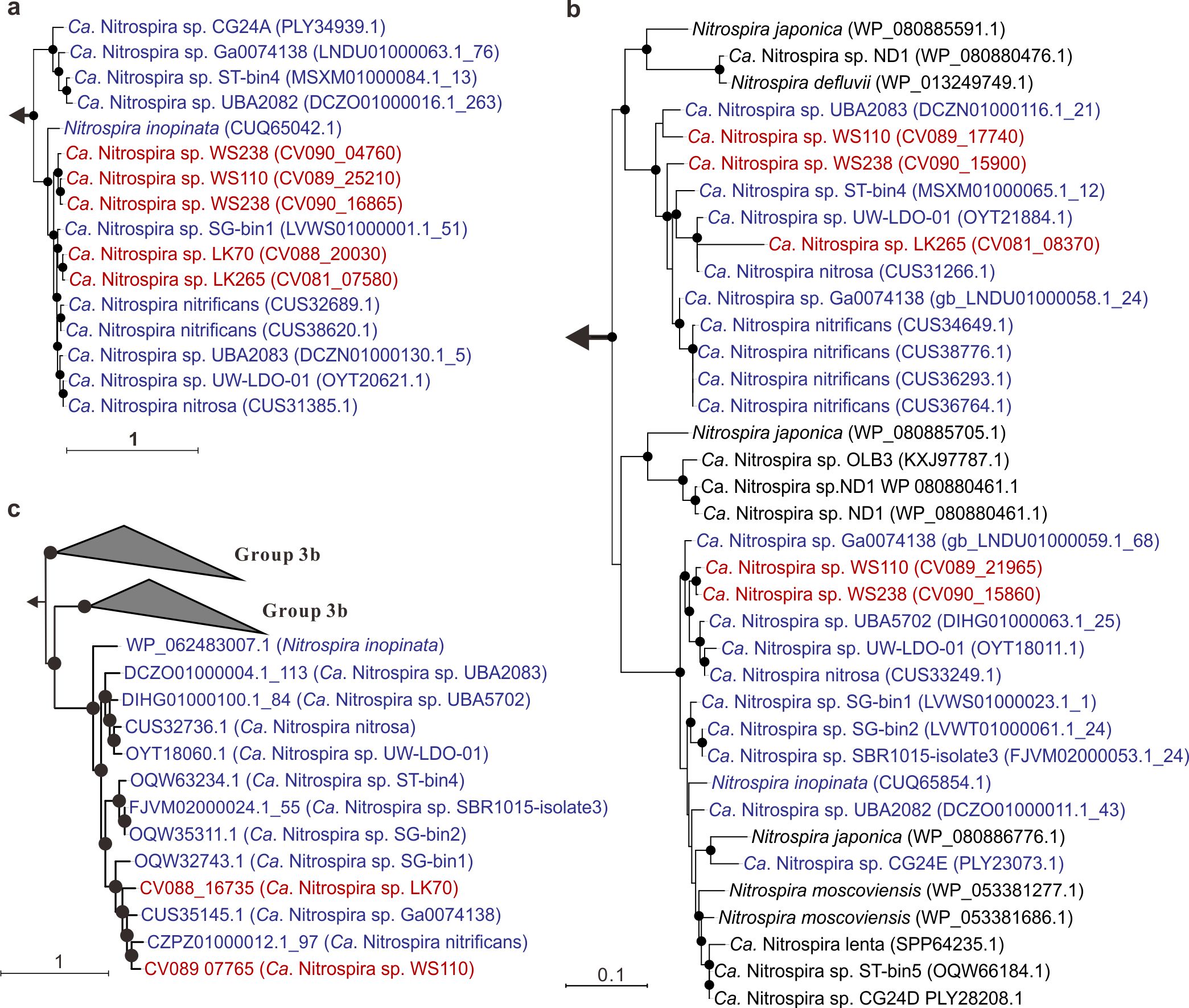

Supplement: FIG S1 [file mBio.03175-19-sf001.jpg]

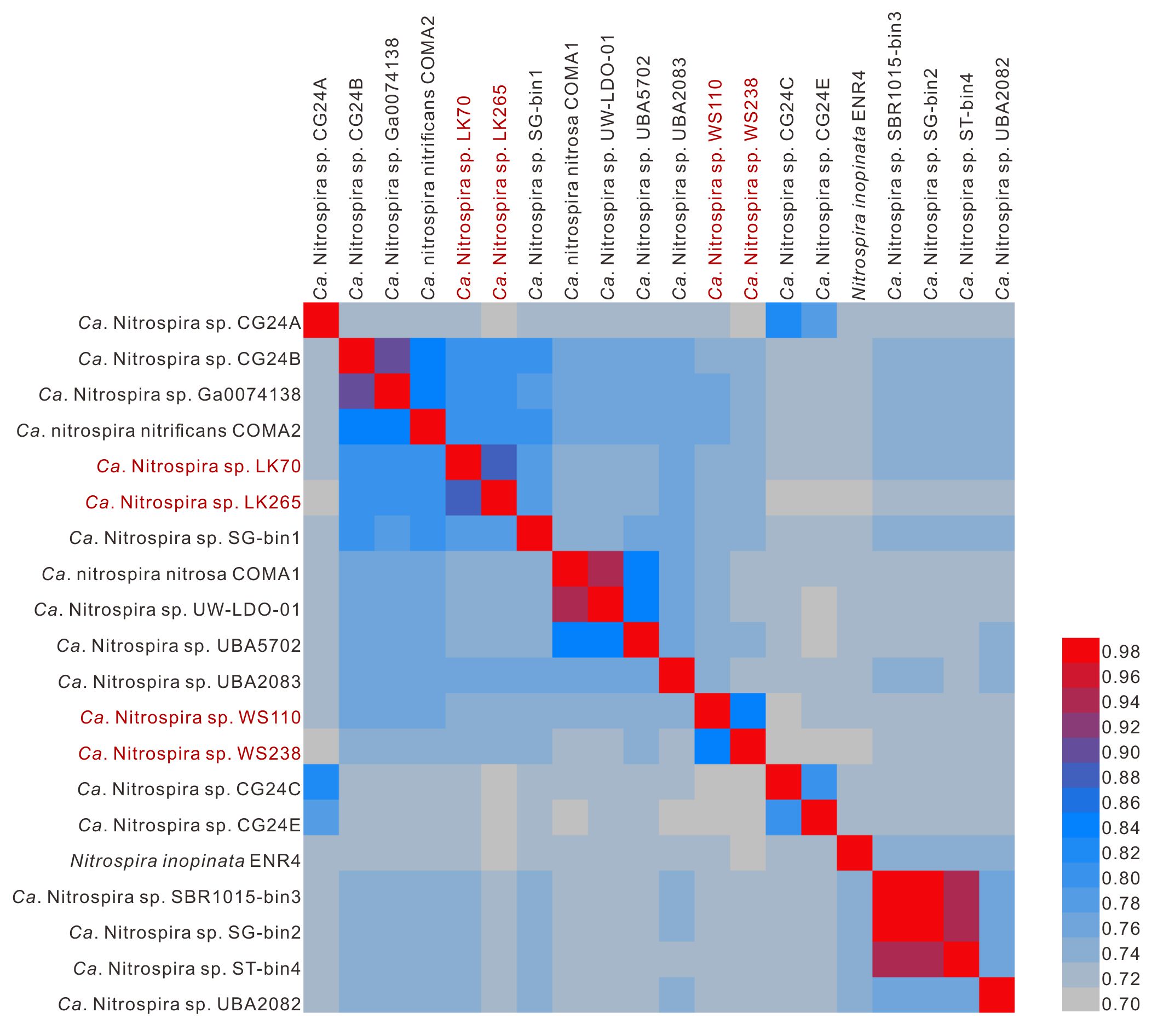

Supplement: FIG S2 [file mBio.03175-19-sf002.jpg]

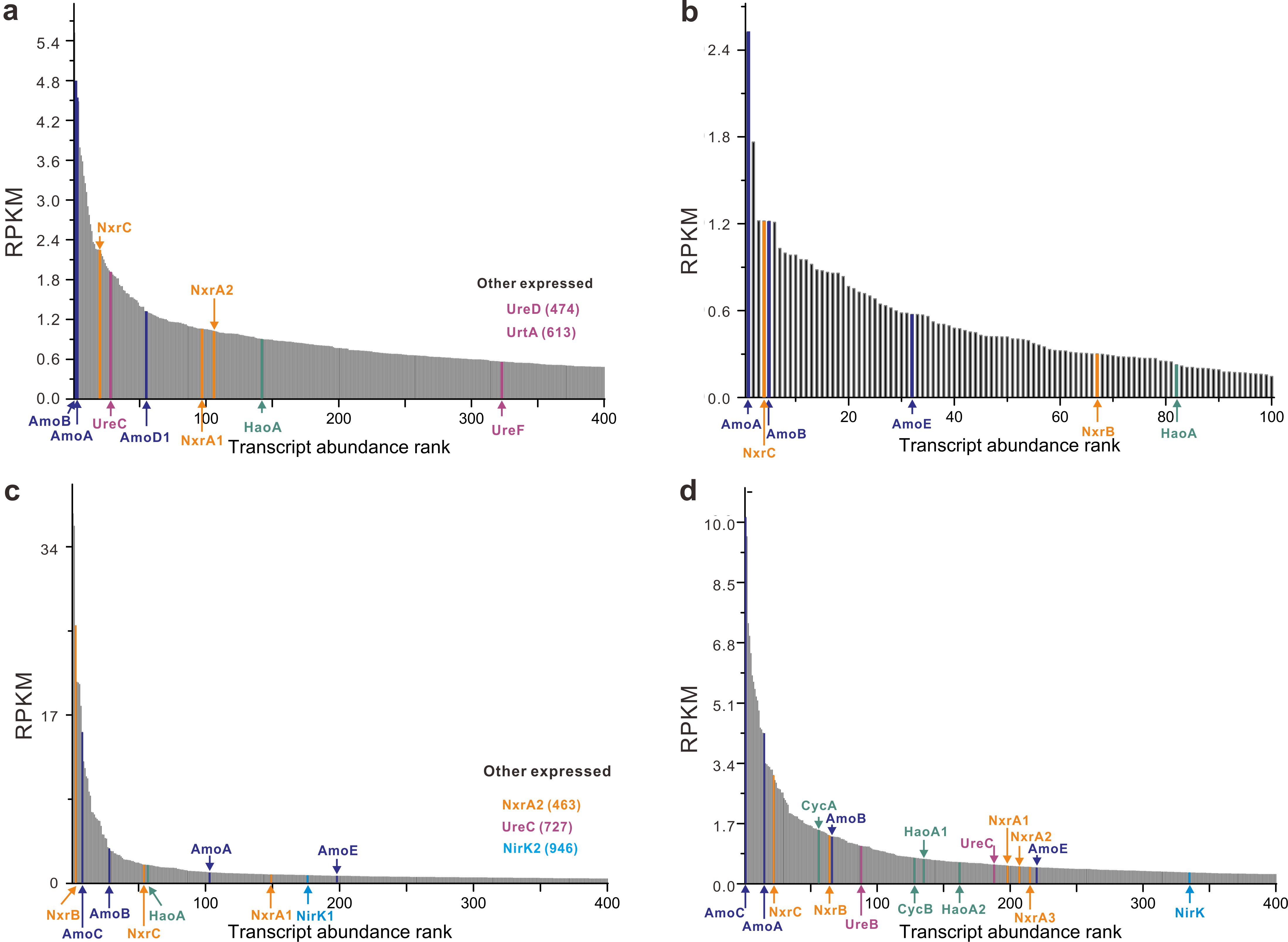

Supplement: FIG S3 [file mBio.03175-19-sf003.jpg]

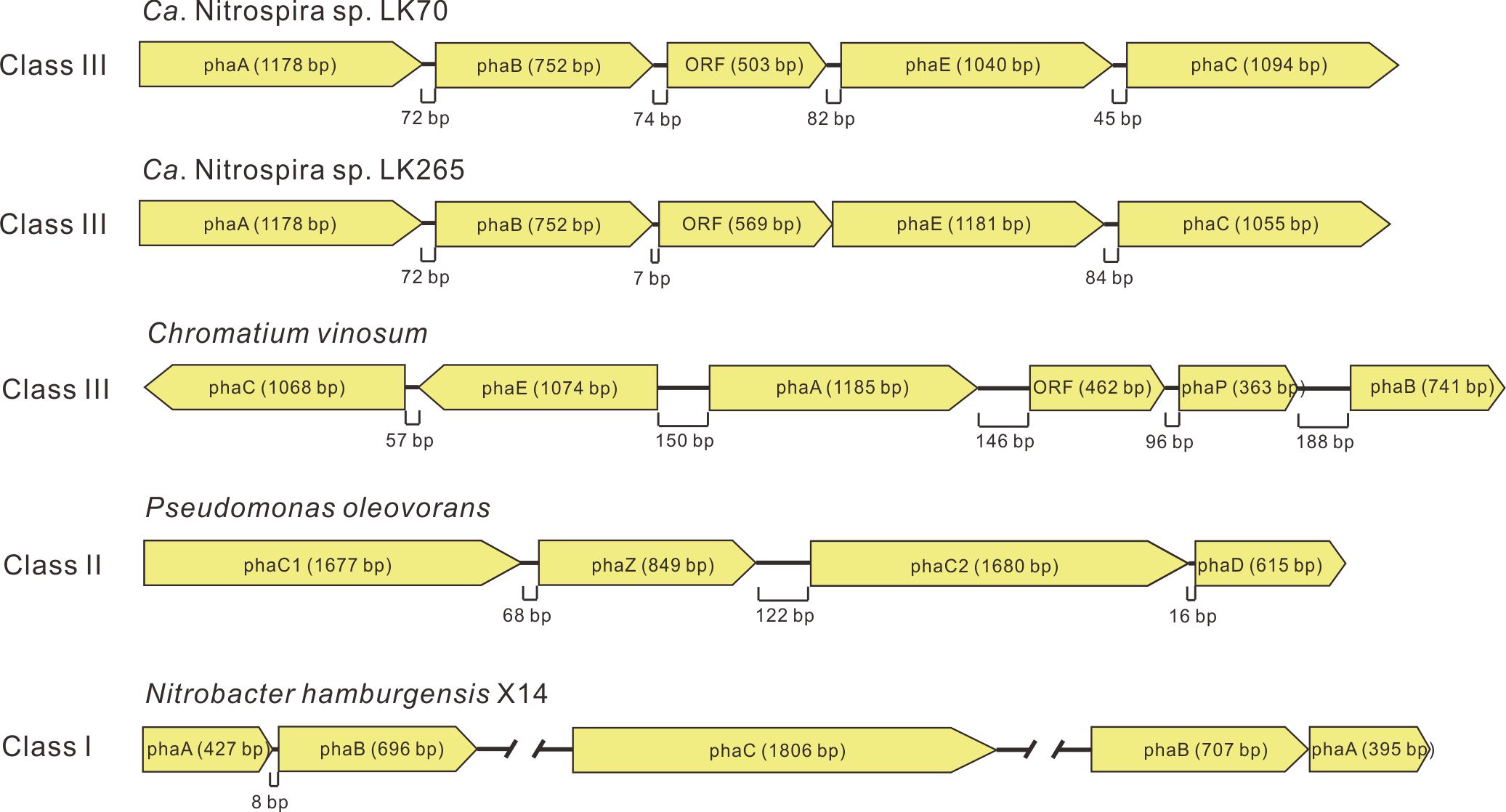

Supplement: FIG S4 [file mBio.03175-19-sf004.jpg]

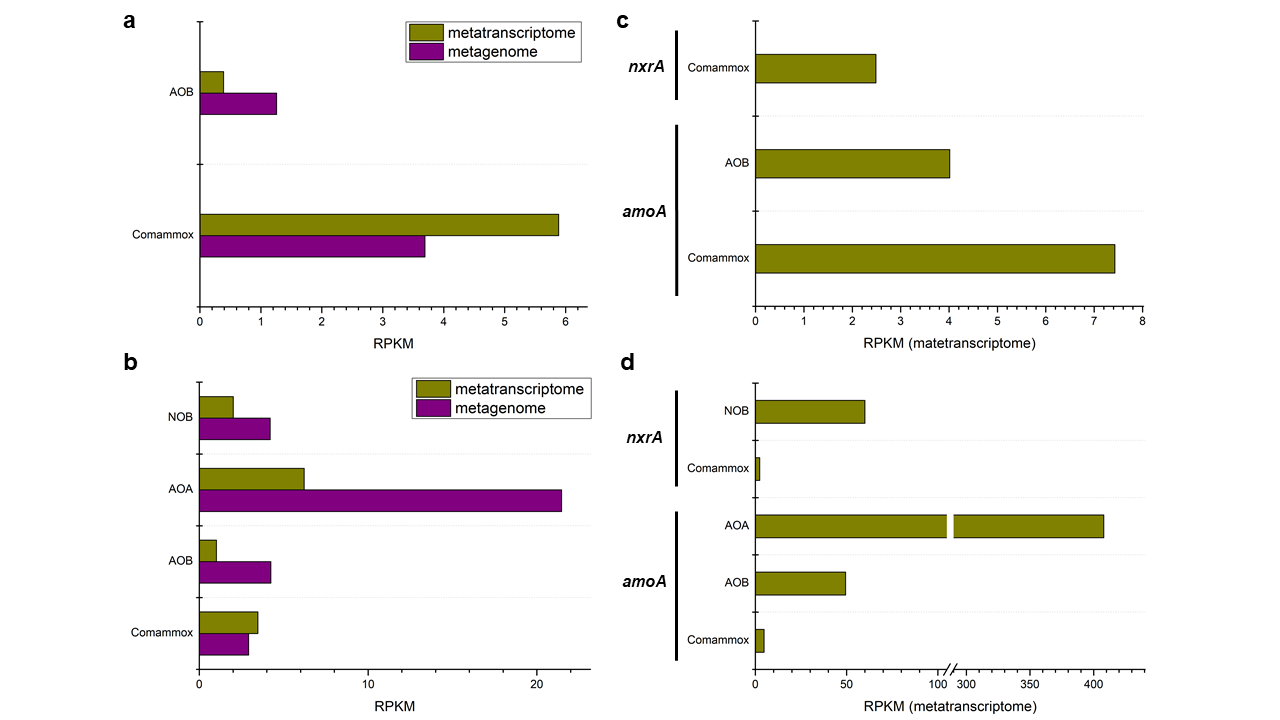

Supplement: FIG S5 [file mBio.03175-19-sf005.tif]

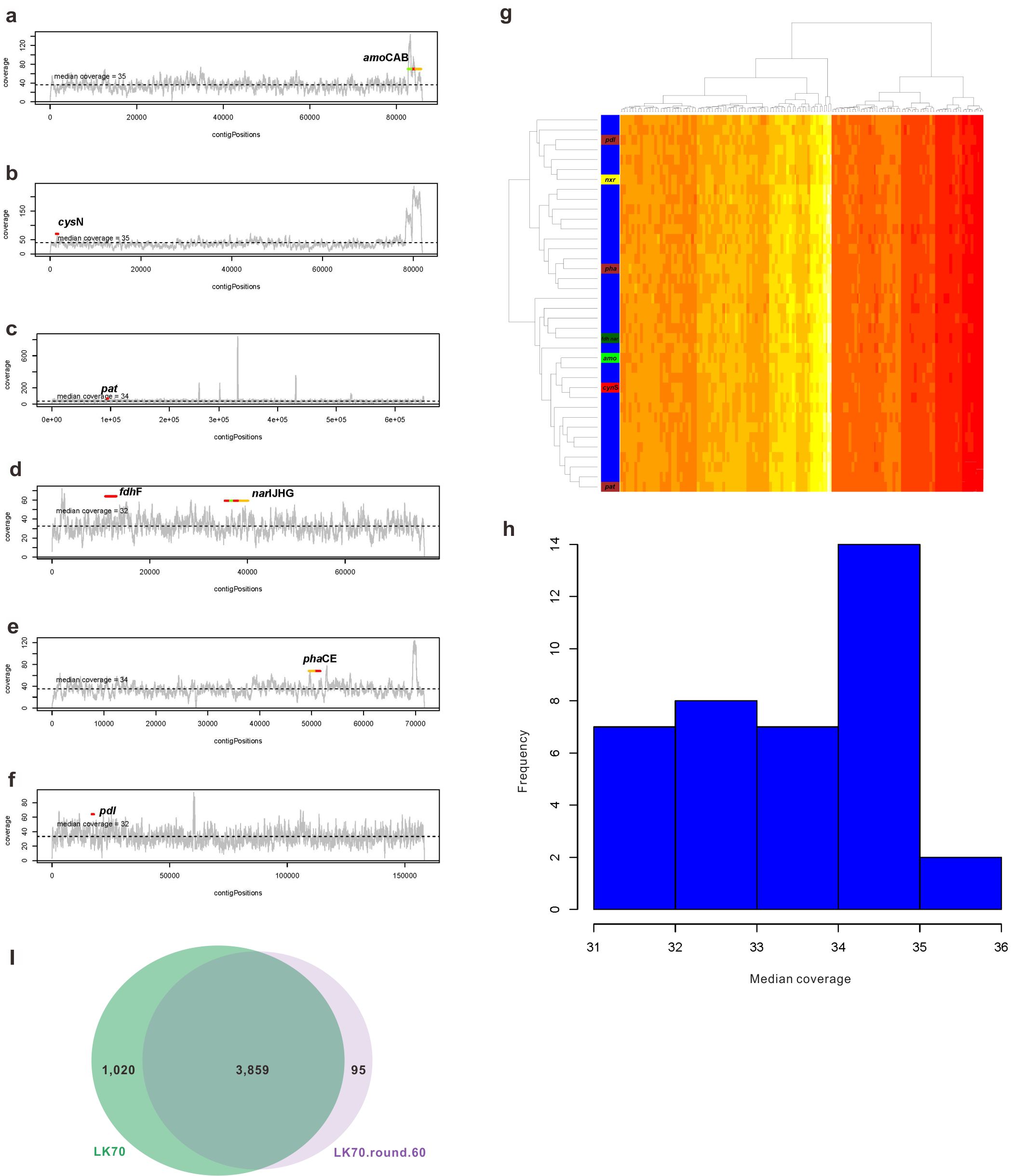

Supplement: FIG S6 [file mBio.03175-19-sf006.jpg]
