# Supplementary material for: Activity and Metabolic Versatility of Complete Ammonia Oxidizers in Full-Scale Wastewater Treatment Systems
Source: mBio. 2020 Mar 17;11(2):e03175-19. doi: 10.1128/mBio.03175-19 (PMC7078480; doi:10.1128/mBio.03175-19)
Supplement: TABLE S1 [file mBio.03175-19-st001.doc]

**Table S1** Quality parameters of the comammox *Nitrospira* genome LK70 after each round of iterative assembly. Comp., completeness; Cont., contamination.

| Bin Id | Comp. (%) | Cont. (%) | Size (Mbp) | No. of scaffolds | N50 (scaffolds) | Mean scaffold length (bp) | Longest scaffold (bp) | No. of predicted genes |
| --- | --- | --- | --- | --- | --- | --- | --- | --- |
| LK70.round.0 | 93.58 | 5.05 | 4.70 | 201 | 37343 | 23375 | 120942 | 4868 |
| LK70.round.1 | 93.58 | 3.23 | 4.57 | 106 | 64724 | 43112 | 239050 | 4628 |
| LK70.round.2 | 93.12 | 3.23 | 4.44 | 80 | 77189 | 55475 | 262614 | 4472 |
| LK70.round.3 | 92.22 | 3.23 | 4.35 | 62 | 94808 | 70090 | 441585 | 4356 |
| LK70.round.4 | 87.67 | 2.32 | 4.27 | 55 | 109077 | 77608 | 355957 | 4265 |
| LK70.round.5 | 87.67 | 2.32 | 4.12 | 50 | 106953 | 82302 | 390901 | 4097 |
| LK70.round.6 | 86.31 | 2.32 | 4.10 | 53 | 98278 | 77380 | 313071 | 4092 |
| LK70.round.7 | 86.31 | 2.32 | 4.11 | 50 | 98278 | 82135 | 405248 | 4090 |
| LK70.round.8 | 86.31 | 2.32 | 4.11 | 48 | 106953 | 85678 | 405248 | 4088 |
| LK70.round.9 | 86.31 | 2.32 | 4.11 | 48 | 106953 | 85726 | 316391 | 4088 |
| LK70.round.10 | 87.22 | 2.32 | 4.12 | 48 | 106953 | 85818 | 316899 | 4093 |
| LK70.round.11 | 87.22 | 2.32 | 4.12 | 47 | 106953 | 87733 | 317317 | 4092 |
| LK70.round.12 | 86.31 | 2.32 | 4.08 | 43 | 110425 | 94994 | 632402 | 4048 |
| LK70.round.13 | 86.31 | 2.32 | 4.02 | 41 | 130584 | 97957 | 632868 | 4002 |
| LK70.round.14 | 86.31 | 2.32 | 4.02 | 41 | 130826 | 98033 | 633092 | 4007 |
| LK70.round.15 | 86.31 | 2.32 | 4.02 | 41 | 131047 | 98010 | 633311 | 3997 |
| LK70.round.16 | 86.31 | 2.32 | 3.99 | 39 | 148032 | 102431 | 633465 | 3981 |
| LK70.round.17 | 86.31 | 2.32 | 4.00 | 39 | 148032 | 102468 | 633641 | 3982 |
| LK70.round.18 | 86.31 | 2.32 | 4.00 | 39 | 148032 | 102499 | 633840 | 3983 |
| LK70.round.19 | 86.31 | 2.32 | 4.00 | 39 | 148032 | 102535 | 634061 | 3980 |
| LK70.round.20 | 86.31 | 2.32 | 4.00 | 39 | 148032 | 102546 | 634223 | 3976 |
| LK70.round.21 | 86.31 | 2.32 | 4.00 | 40 | 130924 | 100026 | 634444 | 3982 |
| LK70.round.22 | 86.31 | 2.32 | 4.00 | 39 | 148032 | 102626 | 634449 | 3984 |
| LK70.round.23 | 86.31 | 2.32 | 4.01 | 40 | 148032 | 100141 | 634449 | 3983 |
| LK70.round.24 | 85.4 | 2.32 | 3.95 | 38 | 148032 | 104041 | 634449 | 3935 |
| LK70.round.25 | 85.4 | 2.32 | 3.95 | 39 | 130924 | 101396 | 634449 | 3938 |
| LK70.round.26 | 85.4 | 2.32 | 3.96 | 38 | 148032 | 104116 | 634449 | 3935 |
| LK70.round.27 | 85.4 | 2.32 | 3.96 | 38 | 148032 | 104122 | 634449 | 3943 |
| LK70.round.28 | 85.4 | 2.32 | 3.96 | 38 | 148032 | 104133 | 634449 | 3941 |
| LK70.round.29 | 85.4 | 2.32 | 3.96 | 39 | 130924 | 101480 | 634449 | 3943 |
| LK70.round.30 | 85.4 | 2.32 | 3.96 | 38 | 148032 | 104192 | 634449 | 3946 |
| LK70.round.31 | 85.4 | 2.32 | 3.96 | 38 | 148032 | 104190 | 634449 | 3940 |
| LK70.round.32 | 85.4 | 2.32 | 3.96 | 38 | 148032 | 104200 | 634449 | 3943 |
| LK70.round.33 | 85.4 | 2.32 | 3.96 | 39 | 130924 | 101547 | 634449 | 3946 |
| LK70.round.34 | 85.4 | 2.32 | 3.96 | 39 | 148032 | 101551 | 415135 | 3947 |
| LK70.round.35 | 85.4 | 2.32 | 3.96 | 39 | 148032 | 101584 | 415185 | 3946 |
| LK70.round.36 | 85.4 | 2.32 | 3.96 | 38 | 148032 | 104252 | 634449 | 3947 |
| LK70.round.37 | 85.4 | 2.32 | 3.96 | 39 | 130924 | 101605 | 634449 | 3944 |
| LK70.round.38 | 85.4 | 2.32 | 3.96 | 38 | 148032 | 104296 | 634449 | 3948 |
| LK70.round.39 | 85.4 | 2.32 | 3.96 | 38 | 148032 | 104321 | 634449 | 3952 |
| LK70.round.40 | 85.4 | 2.32 | 3.96 | 38 | 148032 | 104324 | 634449 | 3958 |
| LK70.round.41 | 85.4 | 2.32 | 3.96 | 38 | 148032 | 104332 | 634449 | 3948 |
| LK70.round.42 | 85.4 | 2.32 | 3.97 | 39 | 130924 | 101671 | 634449 | 3954 |
| LK70.round.43 | 85.4 | 2.32 | 3.97 | 38 | 148032 | 104375 | 634449 | 3950 |
| LK70.round.44 | 85.4 | 2.32 | 3.97 | 38 | 148032 | 104389 | 634449 | 3950 |
| LK70.round.45 | 85.4 | 2.32 | 3.97 | 38 | 148032 | 104397 | 634449 | 3947 |
| LK70.round.46 | 85.4 | 2.32 | 3.97 | 39 | 130924 | 101728 | 634449 | 3956 |
| LK70.round.47 | 85.4 | 2.32 | 3.97 | 38 | 148032 | 104429 | 634449 | 3949 |
| LK70.round.48 | 85.4 | 2.32 | 3.97 | 38 | 148032 | 104424 | 634449 | 3953 |
| LK70.round.49 | 85.4 | 2.32 | 3.97 | 38 | 148032 | 104424 | 634449 | 3951 |
| LK70.round.50 | 85.4 | 2.32 | 3.97 | 39 | 130924 | 101747 | 634449 | 3959 |
| LK70.round.51 | 85.4 | 2.32 | 3.97 | 38 | 148032 | 104447 | 634449 | 3952 |
| LK70.round.52 | 85.4 | 2.32 | 3.97 | 38 | 148032 | 104438 | 634449 | 3955 |
| LK70.round.53 | 85.4 | 2.32 | 3.97 | 38 | 148032 | 104432 | 634449 | 3949 |
| LK70.round.54 | 85.4 | 2.32 | 3.97 | 39 | 130924 | 101752 | 634449 | 3951 |
| LK70.round.55 | 85.4 | 2.32 | 3.97 | 38 | 148032 | 104447 | 634449 | 3956 |
| LK70.round.56 | 85.4 | 2.32 | 3.97 | 38 | 148032 | 104438 | 634449 | 3956 |
| LK70.round.57 | 85.4 | 2.32 | 3.97 | 38 | 148032 | 104432 | 634449 | 3955 |
| LK70.round.58 | 85.4 | 2.32 | 3.97 | 39 | 130924 | 101752 | 634449 | 3959 |
| LK70.round.59 | 85.4 | 2.32 | 3.97 | 38 | 148032 | 104447 | 634449 | 3953 |
| LK70.round.60 | 85.4 | 2.32 | 3.97 | 38 | 148032 | 104438 | 634449 | 3955 |
