# Supplementary material for: Activity and Metabolic Versatility of Complete Ammonia Oxidizers in Full-Scale Wastewater Treatment Systems
Source: mBio. 2020 Mar 17;11(2):e03175-19. doi: 10.1128/mBio.03175-19 (PMC7078480; doi:10.1128/mBio.03175-19)
Supplement: TABLE S2 [file mBio.03175-19-st002.doc]

**Table S2** General features and sources of comammox *Nitrospira* genomes (completeness >85%). Abbreviations: Comp., completeness; Cont., contamination; Comx, comammox; Ref., reference; WWTP, wastewater treatment plant.

| **Group** | **Genome** | **Comp. (%)** | **Cont. (%)** | **Size (Mbp)** | **G+C content (%)** | **No. of scaffolds** | **Coding density** | **Accession number (GenBank or JGI)** | **Sample source** | **Ref.** |
| --- | --- | --- | --- | --- | --- | --- | --- | --- | --- | --- |
| Comx  clade A | *Ca*. N. sp. LK70 | 93.58 | 5.05 | 4.5 | 56 | 202 | 0.87 | PHGA00000000 | WWTP | This study |
|  | *Ca*. N. sp. LK265 | 65.27 | 3.69 | 2.4 | 55 | 492 | 0.88 | PHGB00000000 | WWTP |  |
|  | *Ca*. N. sp. WS110 | 93.53 | 5.50 | 4.5 | 55 | 308 | 0.87 | PHGC00000000 | WWTP |  |
|  | *Ca*. N. sp. WS238 | 66.46 | 16.57 | 3.0 | 55 | 516 | 0.89 | PHGD00000000 | WWTP |  |
|  | *N. inopinata* ENR4 | 96.82 | 4.77 | 3.3 | 59 | 1 | 0.89 | NZ_LN885086.1 | Hot groundwater biofilm | (1) |
|  | *Ca*. N. nitrosa isolate COMA1 | 96.76 | 2.27 | 4.4 | 55 | 15 | 0.87 | NZ_CZQA00000000.1 | Biofilm of aquaculture system biofilter | (2) |
|  | *Ca*. N. nitrificans isolate COMA2 | 96.76 | 2.73 | 4.1 | 57 | 36 | 0.87 | NZ_CZPZ00000000.1 |  |  |
|  | *Ca*. N. sp. SG-bin1 | 95.85 | 3.69 | 4.4 | 56 | 48 | 0.87 | LVWS00000000.1 | Drinking water | (3) |
|  | *Ca*. N. sp. SG-bin2 | 95.85 | 3.69 | 3.7 | 57 | 63 | 0.88 | LVWT00000000.1 |  |  |
|  | *Ca*. N. sp. ST-bin4 | 93.00 | 4.60 | 2.9 | 57 | 117 | 0.87 | MSXM00000000.1 |  |  |
|  | *Ca*. N. sp. UW-LDO-01 | 95.80 | 3.64 | 3.9 | 55 | 230 | 0.86 | NIUT00000000.1 | WWTP | (4) |
|  | *Ca*. N. sp. Ga0074138 | 87.67 | 2.83 | 4.1 | 55 | 66 | 0.87 | LNDU00000000.1 | Drinking water | (5) |
|  | *Ca*. N. sp. UBA2083 | 87.32 | 2.78 | 4.4 | 55 | 137 | 0.84 | DCZN00000000.1 | Sediment | (6) |
|  | *Ca*. N. sp. UBA5702 | 94.09 | 3.86 | 4.3 | 55 | 109 | 0.85 | DIHG00000000.1 | Sediment |  |
|  | *Ca*. N. sp. UBA2082 | 93.18 | 2.78 | 3.6 | 58 | 37 | 0.88 | DCZO00000000.1 | Sediment |  |
|  | *Ca*. N. sp. SBR1015-isolate3 | 95.85 | 3.67 | 3.7 | 57 | 63 | 0.88 | FJVM00000000.2 | Drinking water | (3) |
|  | *Ca*. N. sp. CG24B | 92.22 | 4.65 | 3.2 | 55 | 36 | 0.88 | NEWS00000000.1 | Sand filter biofilm | (7) |
| Comx  clade B | *Ca*. N. sp. CG24E | 93.07 | 3.18 | 3.5 | 56 | 63 | 0.86 | NEWP00000000.1 |  |  |
|  | *Ca*. N. sp. CG24C | 93.05 | 2.73 | 3.0 | 56 | 28 | 0.86 | NEWR00000000.1 |  |  |
|  | *Ca*. N. sp. CG24A | 87.22 | 4.09 | 3.6 | 56 | 58 | 0.85 | NEWT00000000.1 |  |  |

**REFERENCES**

1. Daims H, Lebedeva EV, Pjevac P, Han P, Herbold C, Albertsen M, Jehmlich N, Palatinszky M, Vierheilig J, Bulaev A, Kirkegaard RH, von Bergen M, Rattei T, Bendinger B, Nielsen PH, Wagner M. 2015. Complete nitrification by *Nitrospira* bacteria. Nature 528:504-509.

2. van Kessel MA, Speth DR, Albertsen M, Nielsen PH, Op den Camp HJ, Kartal B, Jetten MS, Lucker S. 2015. Complete nitrification by a single microorganism. Nature 528:555-559.

3. Wang Y, Ma L, Mao Y, Jiang X, Xia Y, Yu K, Li B, Zhang T. 2017. Comammox in drinking water systems. Water Res 116:332-341.

4. Camejo PY, Santo Domingo J, McMahon KD, Noguera DR. 2017. Genome-enabled insights into the ecophysiology of the comammox bacterium "*Candidatus* Nitrospira nitrosa". mSystems 2:e00059-17.

5. Pinto AJ, Marcus DN, Ijaz UZ, Bautista-de lose Santos QM, Dick GJ, Raskin L. 2016. Metagenomic evidence for the presence of comammox *Nitrospira*-like bacteria in a drinking water system. mSphere 1:e00054-15.

6. Parks DH, Rinke C, Chuvochina M, Chaumeil P-A, Woodcroft BJ, Evans PN, Hugenholtz P, Tyson GW. 2017. Recovery of nearly 8,000 metagenome-assembled genomes substantially expands the tree of life. Nat Microbiol 2:1533.

7. Palomo A, Pedersen AG, Fowler SJ, Dechesne A, Sicheritz-Pontén T, Smets BF. 2018. Comparative genomics sheds light on niche differentiation and the evolutionary history of comammox *Nitrospira*. ISME J 12:1779-1793.
