# Supplementary material for: Activity and Metabolic Versatility of Complete Ammonia Oxidizers in Full-Scale Wastewater Treatment Systems
Source: mBio. 2020 Mar 17;11(2):e03175-19. doi: 10.1128/mBio.03175-19 (PMC7078480; doi:10.1128/mBio.03175-19)
Supplement: TEXT S1 [file mBio.03175-19-s0001.docx]

**Supplementary Information**

**Activity and metabolic** **versatility of complete ammonia oxidizers in full-scale wastewater treatment systems**

Yuchun Yang^1,2,4^, Holger Daims^4,5^, Yang Liu^2^, Craig W. Herbold^4^, Petra Pjevac^4,6^, Jih-Gaw Lin^3^, Meng Li^2*^, Ji-Dong Gu^1*^

^1^ Laboratory of Environmental Microbiology and Toxicology, School of Biological Sciences, The University of Hong Kong, Pokfulam Road, Hong Kong SAR, Hong Kong, People’s Republic of China

^2^ Institute for Advanced Study, Shenzhen University, Shenzhen 518060, People’s Republic of China

^3^ Institute of Environmental Engineering, National Chiao Tung University, 1001 University Road, Hsinchu City 30010, Taiwan, People’s Republic of China

^4^ University of Vienna, Center of Microbiology and Environmental Systems Science, Division of Microbial Ecology, Althanstrasse 14, 1090 Vienna, Austria

^5^ University of Vienna, The Comammox Research Platform, Austria

^6^ Joint Microbiome Facility of the Medical University of Vienna and the University of Vienna, Vienna, Austria

*Corresponding author

Meng Li Tel.: (+86) 755-26979250; e-mail: [limeng848@szu.edu.cn](mailto:limeng848@szu.edu.cn)

Ji-Dong Gu Tel.: (+852) 2299-0605; fax: (+852) 2559-9114; e-mail: [jdgu@hku.hk](mailto:jdgu@hku.hk)

**Supplemental Notes:** Core genes of complete ammonia oxidation

**Supporting Information:** Novel comammox MAG LK70 reassembly

**Reference**

**Supplementary Figures**

**Supplementary Tables**

## Core genes of complete ammonia oxidation

One copy of *amoA* and *amoB* was found in each of the four MAGs, whereas three *amoC* copies were identified in LK70, WS110, and WS238. *AmoA* and *amoB* were among the most highly transcribed genes in both LK70 (Fig. S3a) and LK265 (Fig. S3b), but transcript of *amoC* was not detected. In contrast, *amoC* had the highest transcription levels of all *amo* genes in WS110 (Fig. S3c) and WS238 (Fig. S3d). Comparable to previous studies ([1-3](#_ENREF_1)), each of the four comammox MAGs contains components of the *haoAB*-*cycAB* gene cluster that forms the hydroxylamine ubiquinone redox module (HURM) ([4](#_ENREF_4)), although a complete HURM locus was identified only in LK70 and WS238 (Table S3). In addition, WS238 possesses a duplicated *haoA* gene like “*Ca*. N. nitrificans” ([3](#_ENREF_3)). *HaoA* was also highly transcribed (including both *haoA* copies in WS238), but transcripts of *cycA* and *cycB* were detected in WS238 only (Fig. S3d). The comammox MAGs contain one to five copies of *nxrA*, which is the substrate-binding site of NXR ([5](#_ENREF_5)), none to three copies of the electron-channeling subunit *nxrB*, and one copy of the putative membrane anchor of NXR (*nxrC*) (Table S3). The apparent absence of *nxrB* from LK70 is likely due to incomplete genome recovery. Transcription of the *nxrABC* genes was detected for all MAGs (except for *nxrB* in LK70 and *nxrA* in LK265) (Figs. S3a to S3d).

The four comammox MAGs, as well as all other comammox and canonical *Nitrospira* representatives whose genomes have been (almost) completely sequenced ([6-10](#_ENREF_6)), encode copper-dependent nitrite reductase (NirK) (Table S3). The role of NirK in *Nitrospira* remains unknown. Nitric oxide (NO) was recently identified as the actual product of HAO and thus as an important intermediate of bacterial ammonia oxidation ([11](#_ENREF_11)). An enzyme that would oxidize NO further to nitrite has not been identified yet, but NirK operating in reverse has been discussed as one candidate for this function in AOB ([11](#_ENREF_11)). In NOB (*Nitrobacter*), NO produced by NirK was proposed to act as a regulator of the electron flux from nitrite oxidation towards aerobic respiration or towards CO_2_ fixation and storage compound biosynhesis, respectively ([12](#_ENREF_12)). The *nirK* gene was transcribed by the comammox organisms WS110 and WS238 (Figs. 2, S3c, S3d). The role of NirK in comammox and canonical nitrite-oxidizing *Nitrospira* requires further investigation.

## Novel comammox MAG LK70 reassembly

Metagenomic reads were mapped to comammox MAG LK70 with a minimum identity of 98% using BBMap v. 36.32 ([13](#_ENREF_13)). Read pairs for which both reads mapped as well as read pairs for which only one read mapped were extracted from the quality-filtered metagenomic reads and used for re-assembly as a single genome with SPAdes v. 3.10.1 ([14](#_ENREF_14)) using contigs larger than 2 kb from the initial bin with the setting 'trusted-contigs'. Reassembled contigs with inconsistent tetranucleotide composition and sequencing coverage were removed (see “Consistency” explanation below) and the mapping/reassembly procedure was repeated until MAG quality parameters stabilized (see next paragraph and Table S1). This procedure, which allows for a gradual inclusion of additional raw reads, extension of contigs and gap closure as well as a targeted genome-specific removal of contigs that deviate from average composition and/or coverage, has been used previously to generate high-quality MAGs from complex metagenomes ([15-17](#_ENREF_15)).

Consistency between iterations in terms of composition was based on a null model constructed by calculating distance between log-transformed tetranucleotide profiles of randomly selected subsequences of length 2 kb, followed by scaled and centered ordination with principle coordinates analysis (PCoA) in R (prcomp(center=TRUE,scale=TRUE)). This results in a distribution of contig subsequences around a multidimensional origin. The observed distances of 2 kb subsequences were then used to model a normal distribution of distance from origin. Tetranucleotide frequencies from scaffolds were then used in a second PCoA and distances from the origin of complete scaffolds were compared to the null distribution calculated from the 2 kb subsequences to calculate a p-value using pnorm(lower.tail=FALSE) in R and adjusted for multiple testing using p.adjust(“BH”). Contigs were rejected if the adjusted p-value was less than 0.01, indicating a large distance from the origin, and removed for the next iteration. Consistency in terms of coverage was based on mapped reads with a minimum identity of 98% using BBMap v. 36.32 ([13](#_ENREF_13)). Coverage profiles for each scaffold were calculated using samtools ([18](#_ENREF_18)). Coverage profiles were used to construct a null model of coverage by calculating the median coverage of a random selection of 2 kb fragments from the genome. Median coverage of whole scaffolds were then compared to the null model using pnorm() in R to generate p-values. P-values were adjusted for multiple testing using p.adjust(“BH”) after converting high cumulative p-values (>0.995) to right-tail probabilities (prob_right tail­_ = 1-prob­­­­­­_cumulative_). Contigs were rejected if the adjusted p-value was less than 0.005 (left tail or right tail), indicating a large deviation from the median coverage, and removed for the next iteration.

MAG quality parameters were calculated using CheckM ([19](#_ENREF_19)) after every 10 iterations and evaluated for using cor.test(x=iteration#, y=parameter) in R. No MAG quality parameter showed a significant change between iteration 50 and 60 and a high-pure re-assembly LK70.round.60 was acquired (Table S1). Tetranucleotide compositions and sequencing coverage of all scaffolds in the reassembled bin LK70.round.60 were calculated and visualized by house-keeping R scripts to evaluate the assembly quality. Tetranucleotide compositions (Fig. S6g) and sequencing coverage (Fig. S6h) of scaffolds in LK70.round.60 are highly consistent with each other. Average nucleotide identity (ANI) and average amino acid identity (AAI) between LK70 and reassembled bin LK70.round.60 were calculated by OrthoANI ([20](#_ENREF_20)) and AAI calculator ([21](#_ENREF_21)), respectively, with the default settings. ANI and AAI between these two MAGs are 99.92% and 99.91%, respectively. Comparisons of the gene contents between the two assemblies were analyzed by reciprocal best BLAST. Most of predicted genes in reassembled LK70.round.60 were encoded by LK70 (Fig. S6i). But because of the reduced completeness during the reassembly (Table S1), a large number of genes in LK70 could not be found in LK70.round.60 (Fig. S6i) and most of these lost genes have top identities with genes of published comammox *Nitrospira* genomes by searching against NCBI nr database using BLASTP (data not shown). Based on above analyses, it is quite reliable that the new assembly is derived from the same comammox *Nitrospira* genome in the metagenomic dataset. Comammox *Nitrospira* marker gene *amoA* and other novel genes (*cynS*, *pat*, *fdhF*, *phaCE*, and *pdl*) were identified in LK70.round.60 with the similar sequencing coverage and tetranucleotide frequencies with each other and other genes in the same scaffold (Fig. S6a-f). The sequencing coverage of *amoC* is strangely high (Fig. S6a), possibly because the excrescent *amoC* short reads belonging to another two copies *amoC* in the same MAG and another comammox LK265 and AOB (Fig. S5) in the same metagenomic dataset were mapped to LK70.round.60. The reassembly results strongly support that cyanase (*cynS*), formate dehydrogenase (*fdhF*), PHA synthesis (*phaCE*) and degradation (*pdl*) enzymes, and phosphate acetyltransferase (*pat*) in homoacetate fermentation are encoded by comammox strain LK70. The reassembled MAG LK70.round.60 was not submitted to NCBI and used for genome analysis; the highly iterated and rigorous reassembly was only used to exclude the possibility that the novel genes identified in LK70 could be contaminations in binning using Maxbin ([22](#_ENREF_22)).

# REFERENCES

1. Koch H, Lücker S. 2018. Complete nitrification: insights into the ecophysiology of comammox *Nitrospira*. Appl Microbiol Biotechnol doi:10.1007/s00253-018-9486-3:1-13.

2. Daims H, Lebedeva EV, Pjevac P, Han P, Herbold C, Albertsen M, Jehmlich N, Palatinszky M, Vierheilig J, Bulaev A, Kirkegaard RH, von Bergen M, Rattei T, Bendinger B, Nielsen PH, Wagner M. 2015. Complete nitrification by *Nitrospira* bacteria. Nature 528:504-509.

3. van Kessel MA, Speth DR, Albertsen M, Nielsen PH, Op den Camp HJ, Kartal B, Jetten MS, Lucker S. 2015. Complete nitrification by a single microorganism. Nature 528:555-559.

4. Klotz MG, Stein LY. 2008. Nitrifier genomics and evolution of the nitrogen cycle. FEMS Microbiol Lett 278:146-156.

5. Lücker S, Wagner M, Maixner F, Pelletier E, Koch H, Vacherie B, Rattei T, Damsté JSS, Spieck E, Le Paslier D, Daims H. 2010. A *Nitrospira* metagenome illuminates the physiology and evolution of globally important nitrite-oxidizing bacteria. Proc Natl Acad Sci U S A 107:13479-13484.

6. Kits KD, Sedlacek CJ, Lebedeva EV, Han P, Bulaev A, Pjevac P, Daebeler A, Romano S, Albertsen M, Stein LY, Daims H, Wagner M. 2017. Kinetic analysis of a complete nitrifier reveals an oligotrophic lifestyle. Nature 549:269–272.

7. Camejo PY, Santo Domingo J, McMahon KD, Noguera DR. 2017. Genome-enabled insights into the ecophysiology of the comammox bacterium "*Candidatus* Nitrospira nitrosa". mSystems 2:e00059-17.

8. Palomo A, Pedersen AG, Fowler SJ, Dechesne A, Sicheritz-Pontén T, Smets BF. 2018. Comparative genomics sheds light on niche differentiation and the evolutionary history of comammox *Nitrospira*. ISME J 12:1779-1793.

9. Koch H, Lücker S, Albertsen M, Kitzinger K, Herbold C, Spieck E, Nielsen PH, Wagner M, Daims H. 2015. Expanded metabolic versatility of ubiquitous nitrite-oxidizing bacteria from the genus *Nitrospira*. Proc Natl Acad Sci U S A 112:11371-11376.

10. Lucker S, Wagner M, Maixner F, Pelletier E, Koch H, Vacherie B, Rattei T, Damste JS, Spieck E, Le Paslier D, Daims H. 2010. A *Nitrospira* metagenome illuminates the physiology and evolution of globally important nitrite-oxidizing bacteria. Proceedings of the National Academy of Sciences of the United States of America 107:13479-84.

11. Caranto JD, Lancaster KM. 2017. Nitric oxide is an obligate bacterial nitrification intermediate produced by hydroxylamine oxidoreductase. Proc Natl Acad Sci U S A 114:8217-8222.

12. Starkenburg SR, Arp DJ, Bottomley PJ. 2008. Expression of a putative nitrite reductase and the reversible inhibition of nitrite‐dependent respiration by nitric oxide in *Nitrobacter winogradskyi* Nb‐255. Environ Microbiol 10:3036-3042.

13. Bushnell B. 2014. BBMap: A fast, accurate, splice-aware aligner.

14. Bankevich A, Nurk S, Antipov D, Gurevich AA, Dvorkin M, Kulikov AS, Lesin VM, Nikolenko SI, Pham S, Prjibelski AD, Pyshkin AV, Sirotkin AV, Vyahhi N, Tesler G, Alekseyev MA, Pevzner PA. 2012. SPAdes: a new genome assembly algorithm and its applications to single-cell sequencing. J Comput Biol 19:455-477.

15. Dyksma S, Bischof K, Fuchs BM, Hoffmann K, Meier D, Meyerdierks A, Pjevac P, Probandt D, Richter M, Stepanauskas R, Mußmann M. 2016. Ubiquitous Gammaproteobacteria dominate dark carbon fixation in coastal sediments. ISME J 10:1939-1953.

16. Mußmann M, Pjevac P, Krüger K, Dyksma S. 2017. Genomic repertoire of the Woeseiaceae/JTB255, cosmopolitan and abundant core members of microbial communities in marine sediments. ISME J 11:1276-1281.

17. Pjevac P, Dyksma S, Goldhammer T, Mujakić I, Koblížek M, Mußmann M, Amann R, Orlić S. 2019. In situ abundance and carbon fixation activity of distinct anoxygenic phototrophs in the stratified seawater lake Rogoznica. Environ Microbiol 21:3896-3908.

18. Li H, Handsaker B, Wysoker A, Fennell T, Ruan J, Homer N, Marth G, Abecasis G, Durbin R. 2009. The Sequence Alignment/Map format and SAMtools. Bioinformatics 25:2078-2079.

19. Parks D, Imelfort M, Skennerton C, Hugenholtz P, Tyson G. 2015. CheckM: assessing the quality of microbial genomes recovered from isolates, single cells, and metagenomes. Genome Res 25:1043-1055.

20. Lee I, Ouk Kim Y, Park S-C, Chun J. 2016. OrthoANI: An improved algorithm and software for calculating average nucleotide identity. Int J Syst Evol Microbiol 66:1100-1103.

21. Rodriguez-R LM, Konstantinidis KTJM. 2014. Bypassing cultivation to identify bacterial species. 9:111-8.

22. Wu Y-W, Tang Y-H, Tringe SG, Simmons BA, Singer SW. 2014. MaxBin: an automated binning method to recover individual genomes from metagenomes using an expectation-maximization algorithm. Microbiome 2:26.

23. Søndergaard D, Pedersen CNS, Greening C. 2016. HydDB: A web tool for hydrogenase classification and analysis. Sci Rep 6:34212.
